# Drug Delivery Challenges and Current Progress in Nanocarrier-Based Ocular Therapeutic System

**DOI:** 10.3390/gels8020082

**Published:** 2022-01-28

**Authors:** Md Habban Akhter, Irfan Ahmad, Mohammad Y. Alshahrani, Alhanouf I. Al-Harbi, Habibullah Khalilullah, Obaid Afzal, Abdulmalik S. A. Altamimi, Shehla Nasar Mir Najib Ullah, Abhijeet Ojha, Shahid Karim

**Affiliations:** 1 School of Pharmaceutical and Population Health Informatics (SoPPHI), DIT University, Dehradun 248009, India; 2 Department of Clinical Laboratory Sciences, College of Applied Medical Sciences, King Khalid University, Abha 62521, Saudi Arabia; irfancsmmu@gmail.com (I.A.); moyahya@kku.edu.sa (M.Y.A.); 3 Department of Medical Laboratory, College of Applied Medical Sciences, Taibah University, Yanbu 46477, Saudi Arabia; alhanouf.ibraahim@gmail.com; 4 Department of Pharmaceutical Chemistry and Pharmacognosy, Unaizah College of Pharmacy, Qassim University, Unaizah 51911, Saudi Arabia; h.abdulaziz@qu.edu.sa; 5 Department of Pharmaceutical Chemistry, College of Pharmacy, Prince Sattam Bin Abdulaziz University, Al-Kharj 11942, Saudi Arabia; o.akram@psau.edu.sa (O.A.); as.altamimi@psau.edu.sa (A.S.A.A.); 6 Department of Pharmacognosy, Faculty of Pharmacy King Khalid University, Abha 62521, Saudi Arabia; shehlanasar2005@gmail.com; 7 Six Sigma Institute of Technology and Science, College of Pharmacy, Rudrapur 263153, India; abhi_pharm1@rediffmail.com; 8 Department of Pharmacology, College of Medicine, King Abdulaziz University, Jeddah 21589, Saudi Arabia; shahid.karim@yahoo.co.in

**Keywords:** ocular therapeutic system, drug delivery, hydrogel, nanomicelles, nanoparticles, implant, microneedle

## Abstract

Drug instillation via a topical route is preferred since it is desirable and convenient due to the noninvasive and easy drug access to different segments of the eye for the treatment of ocular ailments. The low dose, rapid onset of action, low or no toxicity to the local tissues, and constrained systemic outreach are more prevalent in this route. The majority of ophthalmic preparations in the market are available as conventional eye drops, which rendered <5% of a drug instilled in the eye. The poor drug availability in ocular tissue may be attributed to the physiological barriers associated with the cornea, conjunctiva, lachrymal drainage, tear turnover, blood–retinal barrier, enzymatic drug degradation, and reflex action, thus impeding deeper drug penetration in the ocular cavity, including the posterior segment. The static barriers in the eye are composed of the sclera, cornea, retina, and blood–retinal barrier, whereas the dynamic barriers, referred to as the conjunctival and choroidal blood flow, tear dilution, and lymphatic clearance, critically impact the bioavailability of drugs. To circumvent such barriers, the rational design of the ocular therapeutic system indeed required enriching the drug holding time and the deeper permeation of the drug, which overall improve the bioavailability of the drug in the ocular tissue. This review provides a brief insight into the structural components of the eye as well as the therapeutic challenges and current developments in the arena of the ocular therapeutic system, based on novel drug delivery systems such as nanomicelles, nanoparticles (NPs), nanosuspensions, liposomes, in situ gel, dendrimers, contact lenses, implants, and microneedles. These nanotechnology platforms generously evolved to overwhelm the troubles associated with the physiological barriers in the ocular route. The controlled-drug-formulation-based strategic approach has considerable potential to enrich drug concentration in a specific area of the eye.

## 1. Introduction

The eye is a unique, complex, and highly important part of the human body, which is related to various physiological and anatomical barriers. Topical drug instillation in the ocular surface is a popular route for the treatment modalities of eye disorders. However, the poor drug retention and permeation resulting in the erratic bioavailability of drugs is a major concern, which should be considered for addressing effective therapy. The blinking, baseline, and reflex lachrymation, as well as drainage to remove foreign substances, including drugs, rapidly from the surface of the eye, results in suboptimal ocular bioavailability [1]. It has been reported that 90% of eye drops available in the market only supplemented 5% of drug bioavailability, and the rest of the drug washed away through different elimination routes such as tear fluid, nasolacrimal secretion, protein binding, enzymatic degradation, or metabolism by protease, and esterase enzyme. Despite these, numerous physiological barriers play significant roles in poor drug retention, including the blood–retinal barrier and the blood–aqueous barrier, along with the corneal barrier [2].

There are many eye ailments, including conjunctivitis, blepharitis, glaucoma, cataract, diabetic retinopathy, and macular degeneration (age related), which affect the anterior and posterior region of the eye; sometimes, the patient may also lose eyesight. The critical challenge ahead of topical application is to improve the drug holding time, by the rationale design of formulation approach, which, ultimately, results in consistent and uniform drug absorption at the site of application in the eye being indigently addressed. Based on conventional techniques, several types of ophthalmic drug delivery systems are available in the market. Aside from eye drops, other topical preparations available in the market are eye ointments, gels, and ocular inserts such as eye dosage formulations, which tend to somehow increase the holding time of drugs in the eye, but the appearance of blurred vision and the related inconvenience has limited their use. Moreover, the systemically given drug for action in the eye has limited access due to poor blood flow in the corneal cells and tissues. Injection into the eye cavity is sometimes recommended for drug delivery into the posterior region, which is painful and causes patient incompliance. The speedy drainage of the drug from topical application reduces the pharmacological action of the drug, which needs to be compensated with increased dosing frequency as well as the part of the drug that has reached systemic circulation through the various routes that could cause a systemic toxic effect. Indeed, to surmount these problems, novel ophthalmic preparations, viz., NPs, liposomes, prodrug, nanomicelles, nanosuspensions, dendrimers, contact lenses, implants, microneedles, and in situ gel, have promising results, which have been explicated in the last few decades to alleviate better drug solubility, dissolution, absorption, and bioavailability in a controlled, sustained, and prolonged period [1,3].

## 2. Ocular Anatomy

The human eye consists of different layers with specific internal structures, and each part perform performs specific functions.

### 2.1. The Sclera, Cornea, and Conjunctiva

The sclera is the white part of the eye that appears as an opaque, hard white sheath, which comprises the outer layer of the eye ball. It resembles a stiff fibrous membrane that preserves the eye shape. It continues within the cornea and is much thicker towards the posterior part of the eye than the anterior part of the eye. It is composed of collagen fibers and proteoglycans engrafted in the extracellular matrix. The hydrophilic solutes are more permeated through the sclera than the conjunctiva and cornea, due to diffusive transport across the leaky region of collagen fibers. The presence of charges (+/−) on the surface of molecules also influences permeability across the sclera. The cornea is the principal route in the topical drug instillation, while conjunctiva and sclera allow hydrophilic drugs to diffuse through the ciliary body. It gives protective covering, maintains intraocular pressure, and is an attachment site for the ciliary muscles [4,5].

The cornea is clear and transparent, and it lacks the blood supply situated at the most anterior part of the eye. It consists of different parts such as corneal epithelium externally, Bowman’s membrane, corneal stroma, and the endothelial layers internally. The corneal permeability is important in how the drug penetrates and retains in the aqueous humor. The trans-corneal diffusion of lipophilic drugs is mediated via corneal epithelial, which is a rate-limiting barrier for such drugs. The corneal stroma is made up of hydrophilic collagen and causes the hindrance for the diffusion of the lipophilic drugs and is interconnected by several ciliary nerves [6]. The conjunctiva inside the eye is a fragile, slim, and transparent epithelial barrier, which channelizes inward toward the eyelids and comprehends the anterior and one-third of the eyeball part. The conjunctiva is constituted of an upper layer, epithelium cells, and their inherent substantia propria. The conjunctiva contributes to the production of the tear film with substantial electrolytes, fluid, and mucus secretion and thus prevents the microorganism entry and lubricates the eye cavity. The surface area of the conjunctiva is larger than the cornea and is more permeable to the drugs. However, the significant drug loss occurs due to their blood capillaries and lymphatic that is drawn into the systemic circulation. The conjunctival drug absorption is considered futile due to the conjunctival blood cells, capillaries, and tight junctions, leading to excess loss of drug in the systemic circulation, hence resulting in poor ocular drug bioavailability [6].

### 2.2. Aqueous Humor

This is a nonvascular, transparent, clear fluid that occupies the posterior and anterior parts of the eye. It provides essential nutrients such as sodium, chloride ion, ascorbate salt, extricates waste products from the nonvascular tissue to the cornea, and maintains intraocular pressure by controlling Schlemm’s canal and the shape of the cornea. Aqueous humor drains out from Schlemm’s canal, a circular groove that enters there from the anterior chamber and releases into the blood circulation via the anterior ciliary veins. The turnover rate of aqueous humor formation in humans varies 1–1.5% of the anterior chamber volume per minute. The rate of an aqueous humor formation is ~2.5 µL/min [7].

### 2.3. Pupil, Iris, and Ciliary Muscle and Vitreous Humor

**Figure 1:**
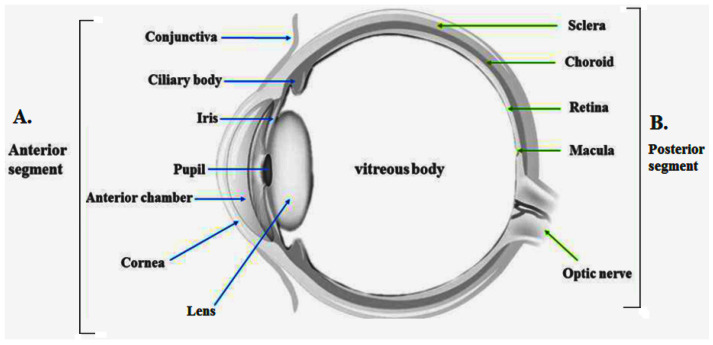
The anatomy of ocular system representing anterior (**A**) and posterior segments (**B**). The anterior segment includes conjunctiva, ciliary body, iris, pupil, anterior chamber, cornea, and lens. The posterior segment consists of sclera, choroid, retina, macula, and optic nerve. Modified from ref. [8]; permission under a creative common license (CC BY-NC-ND 4.0).

The central dark part of the eye is a chamber associated with the passes of light into the eye. Light reflex is a vital phenomenon by which pupil shape and size are modulated by the pupillary reflex. The iris is a circular, muscular contractile structure confronting the lens back to the cornea. The diaphragm of the iris is of variable sizes that work to line up the pupil size and regulate the incident light into the eye. It is a colored part of the eye, ranging from blue to grey, which creates varying visual aspects. The ciliary muscle is present in the middle eye layer, which controls and coordinates eyeshot to the objects with the accommodating distances and controls the aqueous humor flow into the Schlemm’s canal. The ciliary muscle is responsible for contraction or relaxation, which enables the eye to focus near or far objects. The vitreous humor constitutes ~80% of the total volume in each eye in the human body. Physically, it appears as a jelly-like transparent substance present in the chamber back to the eye lens [8]. A detailed schematic expression of the components of each eye part is illustrated below (Figure 1). 

### 2.4. The Retina, Macula, Choroid, and Optic Nerve

The retina is situated at the posterior side of the eye. It consists of photosensitive rod and cone cells, along with the glial, neural, vascular cells, and nerve fibers that convey the light through nerve impulses and extended to the brain via the optic nerve. The macula is located at the center of the retina and contains a vast number of photoreceptor cells that transfer the light into the nerve signals. 

The macula has a noval structure, a pigmented part situated near the central part of the retina, having various ganglion cells and approximately 200 million neurons. The pigment assimilates light and transmits the light signal to the brain through the optic nerve. The optic nerve sends the signals from the eye to the brain, which contains image information for processing by the central nervous system. A thin layer of tissue situated between the sclera and retina is choroids that possess blood vessels that carry oxygen and nutrients to the eye and restrict drug administration into the posterior chamber. The forepart of the optic nerve is called an optic disk [8].

### 2.5. Accessory Parts in Eye

There are several parts of the eye that protect it against injury such as the eyebrows, eyelids, eyelashes, and lacrimal apparatus. The eyebrow gives protection to the anterior part of the eyeball from pollens, dust particles, and foreign bodies. The different layers of tissues in eyelids with conjunctiva protect the fragile cornea and eye front. Upon instillation of eye drops in the lower conjunctival sac, the lachrymal glands release the tear fluid, which contains water, mineral salts, lysozyme, antibodies, and antimicrobial enzymes. The excess of eye drops drains out into the gastrointestinal tract through the nasolacrimal system immediately upon instillation, which may be because either the volume of the drug dose exceeds the volume of lachrymal fluid or reflex tearing. The excess volume moves into the gastrointestinal tract through the nasolacrimal drainage [9,10].

## 3. Constraints in Ocular Drug Delivery

The loss of a drug after instillation in the eye is a major constraint in the ocular therapeutic system, and this may be from the ocular surface, lacrimal fluid secretion, and the blood–ocular barrier.

### 3.1. Precorneal Barrier

After administration of the eye drop, the onset of lacrimal fluid secretion swiftly removes medicaments from the eye surface. Notably, the lacrimal fluid turnover rate is ~1µL/min, and the redundant drug dose fluid is taken off in a short period of time through the nasolacrimal ducts. The precorneal factors including the tear turnover and reflex blinking clearance mechanism due to increased volume of liquid in cul-de-sac largely limited the drug retention in the anterior eye segment. The volume of liquid more than 9 µL may turn into a tear and be drawn out through the nasolacrimal duct [10]. 

### 3.2. Corneal Epithelial Barrier

This is considered a primary barrier to the topical administration of a drug. The diversified layer of columnar and squamous epithelial cells, as well as the intercellular tight junctions, act as permeation barriers of therapeutics through paracellular routes. The occurrence of calcium ion levels and various protein molecules congest the epithelial tight junctions. However, the disruption of the tight junction membrane or the complexation of calcium ions with EDTA somehow improves the drug permeability [11].

### 3.3. Lacrimal Sac Eye Barriers

Approximately 95% of a drug instilled into the eye is eliminated from blood circulation through the nasolacrimal duct. It acts as a conduit for the tear flow into the nasal chamber from the eye. The nasolacrimal drainage system consists of a lacrimal sac, canaliculi, and nasolacrimal ducts. Due to the vascularized wall of the nasolacrimal and lacrimal sac, half of the drug concentration is absorbed there. The constraint due to this also depends on the volume of the topically applied drug solution, patients’ reflex blinking, and age. The drug delivery design should enable it to be retained on the ocular surface to release a sufficient concentration of the drug in the lacrimal fluid [12]. 

### 3.4. Blood–Ocular Barriers

**Figure 2:**
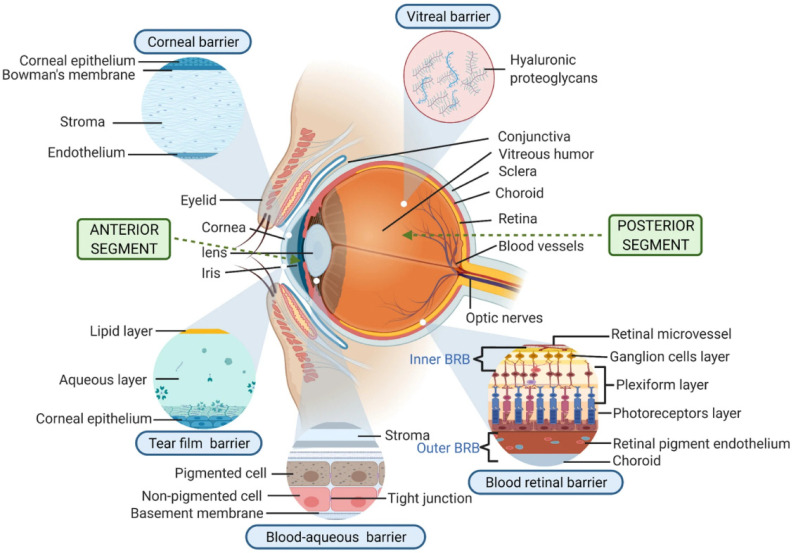
Drug delivery barriers in ocular route. The barriers may be from the anterior segment including corneal barriers, e.g., corneal epithelium, tear film, conjunctiva, and blood–aqueous barrier. The posterior segment barrier may be due to the blood–retinal barriers comprising retinal vessels, ganglion cells, pigment cells, retinal endothelium, and the vitreous barrier. These barriers overall reduced drug availability in the ocular tissues of the posterior segment. Permission received under Creative Commons Attribution 4.0 International License [17].

The blood–ocular barrier encompasses the blood–aqueous barrier (BAB) and the blood–retinal barrier (BRB), which are significant barriers in topical drug delivery in the anterior and posterior chambers of the eye. BRB, related to the anterior chamber of the eye, consists of the ciliary endothelium (nonpigmented) and the ciliary blood vessels/endothelium of the iris. This cell layer expresses the tight junctions of the endothelial cells of the retina and thus restricts the entry of a drug molecule into intraocular surroundings. BRB, however, prevents drug entry from the blood into the posterior chamber. It comprises retinal capillaries, which include the inner retina barrier and the retinal pigment epithelium cells (RPEs), considered as the outer blood–retinal barrier, respectively [13,14]. The drug permeability across RPEs is easier to determine, but it is hard to quantitate the permeability values of the vascular component of the BRB. The particle size is a concern for the permeation of a drug molecule from retinal capillaries. A radio-labeled tracer study revealed that retinal capillaries prevent the penetration of carbon NP of size 20 nm, but small molecules of molecular weight in Dalton (fluorescin and mannitol) were easily permeated. It has also been reported that epithelial tight junction has spaces of 2 nm, and similar size molecules can easily permeate. Another study has also reported that a retinal capillary is a vital barrier that allows only those molecules to permeate with a size of ≤2 nm [15]. The RPE is a tight junction located between choroid and photoreceptors and maintains homeostasis in the neural retina. Studies suggested that the permeability toward RPE is dependent on the lipophilicity and size of the compound. The orally or intravenously (i.v.) administered drug prominently enters the choroids rather than retinal capillaries, due to high vasculature. The choroid capillaries also assist in attaining an equal concentration of drug molecules in blood circulation and in extravascular spaces in choroids and also prevent the drug entry into the retina. The BAB comprises endothelial cells, iris, ciliary muscle, and pigmented and nonpigmented epithelium cells. The tight intercellular junctions are also present in the epithelial components of BAB. The simultaneous elimination of the drug molecules together from BRB and BAR makes it difficult to estimate the drug permeability separately [9]. Therefore, the blood–retinal barrier is still a major gainsay for topical drug delivery into the retina. To overcome the retinal barrier by permeating the inner limiting membrane (ILM), Tavakoliet al. developed PEGylated liposomes for better permeation in the retinal cells. The particle size and surface charge play pivotal roles in retinal permeation; sizes of liposomes >100 nm are betrayed to pass through the ILM, as the study revealed. Smaller sizes of anionic PEGylated liposomes (~50 nm) were found to have excellent distribution and penetration into the retinal cells [16]. An illustrative diagram showing various barriers in the ocular route is presented in Figure 2.

### 3.5. Efflux Protein Barrier

The efflux protein barrier is present in the apical cell membrane of the conjunctiva epithelial cells, nonpigmented ciliary epithelial cells, and retinal endothelial cells. It has control over enhancing or retarding the drug absorption based on cellular localization. The major efflux protein responsible for the drug absorption is P-glycoprotein, which acts as an efflux pump and prevents entry of hydrophilic and lipophilic molecules, in both abnormal and normal cells. P-glycoprotein is also said to be multidrug resistance mutation 1 (MDR1), an ATP-dependent efflux transporter that greatly reduced the drug concentration in multidrug-resistant tumor cells. The multidrug resistance protein (MRP) is also a membrane-bound efflux transporter and is detected in different ocular tissues. Among the different types of efflux transporter investigated by Chen et al., MRP1–4 and MRP6 are located in the corneal epithelium. The expression level of MRP2–4, MRP6, MDR1, and breast cancer resistance protein (BCRP) is detected in the basal cell layer of the human conjunctiva, while MRP1 and MRP7 are expressed in the entire conjunctival epithelium [18]. Zhang et al., investigated drug transporter and cytochrome P450 mRNA expression level in the ocular drug disposition and revealed low levels of BCRP and MRP2 in the human cornea [19]. 

## 4. Conventional Ocular Therapeutic Systems

In the market, surplus ocular/ophthalmic products such as eye drops are available; this covers ~70% or more of prescription drugs available, due to ease of application, noninvasiveness, patient compliance, and cost effectiveness.

### 4.1. Eye Drops

The eye drop is a simple and convenient, noninvasive, patient compliant, and safer way of delivering medicament into the eyes. The drug release from eye drops follows a pulsatile pattern immediately post instillation, and the concentration of the drug rapidly declines thereafter. The drug release from the eye drops follows first-order kinetics. The critical challenge in topical drug delivery is to improve the contact time of therapeutics in the eye, which may enhance the bioavailability of the drugs. To this end, novel additives may be incorporated to increase the viscosity of the formulation, which, in turn, improve the precorneal residence and ocular permeation of drugs. Certain viscosity enhancers such as carboxymethyl cellulose, sodium carboxymethyl cellulose, and hydroxymethylcellulose are used to improve precorneal residence time and bioavailability. For enhancing the permeation rate in cornea cyclodextrin, ion-pairing forming agents and iontophoresis technique are used [20,21]. 

The permeation enhancers including polyoxyethylene glycol ethers, ethylene diamine tetra-acetic acid sodium, Tween 80, span 80, and Brij-35 ameliorate corneal uptake through altering the corneal integrity and physiological environment in the ocular surface [21]. 

### 4.2. Liquid Dosage Form

#### 4.2.1. Emulsions

An emulsion is a biphasic therapeutic system that consists of two immiscible phases; it is conventionally used and still a good option to enhance solubility, dissolution, and drug absorption. For dispensing medicaments in an emulsion vehicle, it is prepared as oil in water (o/w) and water in oil (w/o), which is commercially available for ophthalmic drug delivery. The o/w type emulsion is largely explored in the ocular formulation, compared with the w/o type, due to ocular tolerance, with less or no irritation, in which hydrophobic drugs are blended in the oily phase and aqueous phase thereafter. An example of the ophthalmic eye drop is a cyclosporine-A (Restasis), which comprises 0.05% emulsion of cyclosporine-A and is used in chronic dry eye treatment. Cyclosporine improves tear production by reducing the inflammation in the eye. The azithromycin ophthalmic emulsion (AzaSite^®^) is composed of 1% azithromycin ophthalmic emulsion intended for the purpose of bacterial conjunctivitis and other ocular complications. AzaSite^®^ ophthalmic emulsions are available in the United States. Refresh Endura is a nonmedicated emulsion used for dry eye disease [22]. Many studies quoted in the literature have successfully manifested the relevancy of using emulsions for better precorneal residence, enhanced corneal permeation of the drug, the provision of prolonged or sustained release of medicine, and overall, improved ocular bioavailability.

For delivering medicine into the anterior ocular tissue, Tajika et al., demonstrated the enhanced anti-inflammatory action of 0.05% difluprednate, a prednisolone derivative, using an emulsion as a vehicle. An animal study on rabbit’s revealed that emulsion was successfully delivered into the anterior chamber of the animal eye, with little concentration of the drug transferred to the posterior part of the eye following instillation of the eye drop one or more times in a day [23].

Formulation additives such as lipid soy lecithin and stearyl amine were used as carriers for azithromycin, which showed improved ocular drug absorption and bioavailability. The lipid emulsion of azithromycin was compared with plain drug solution at varying doses of the drugto study the tear elimination feature. An animal study on rabbits revealed that after topical administration of azithromycin, emulsion showed a sustained release, improved stability, and precorneal residence time [24]. 

**Figure 3:**
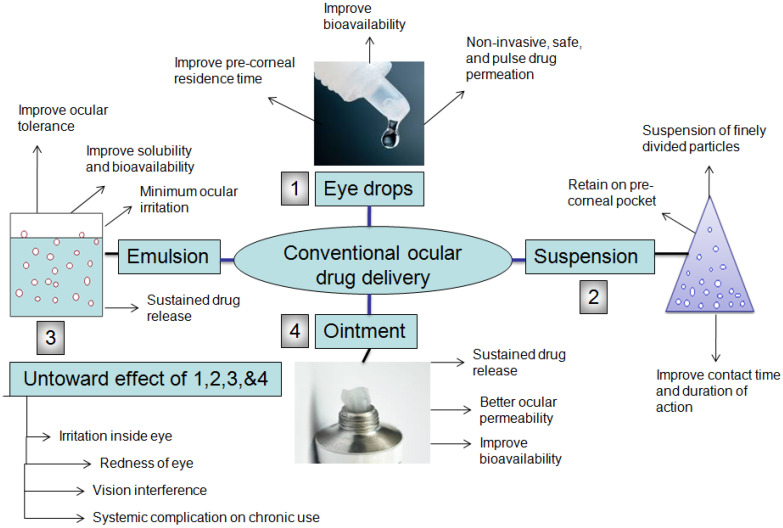
Conventional mode of ocular therapeutic system.

Similarly, with the intent to improve the ocular drug availability using emulsion as a vehicle, Shen et al. developed a flurbiprofen emulsion. The preparation comprised flurbiprofen axetil, castor oil, and tween 80 as oil and surfactant, as well as an aqueous phase. They prepared different emulsions with changing ratios of castor oil (0.1–2.5%) and tween 80 (0.08–4%), which were labeled as formulation F1–F4, respectively. The topical administration of F2 emulsion drop in albino rabbits showed improved pharmacokinetic with castor oil/tween 80 (0.5:0.4) by weight%, compared with other preparations and drug solutions. The F2 emulsion was better translocated and achieved higher concentration of drug in the aqueous humor, compared with a 0.03% flurbiprofen sodium eye drop [25]. The conventional therapeutic approach in ocular delivery is shown in Figure 3.

#### 4.2.2. Suspensions

Suspension is a biphasic liquid preparation method in which API is finely dispersed in a dispersion medium or aqueous solvent essentially composed of a suspending agent and a suitable dispersing agent. In other words, suspensions are API-saturated carrier systems. The application of the eye drop suspension is inevitable for hydrophobic drugs due to limited aqueous solubility. The drug in suspension state retains longer in cul-de-sac cavity and ocular tissue, thereby increasing the ocular residence time and bioavailability and efficacy of the drug, compared with eye drop solutions [26]. The particle size has a crucial role in drug effectiveness in suspensions—larger particles will take more time to dissolve and may show prolonged retention time; on the other hand, smaller particle sizes are easily absorbed into ocular tissues from precorneal spaces. As regards patient compliance, the particle should be kept below 10 microns in eye drop suspensions. Notably, in addition to particle size, at the same time, the surface morphology of suspended particles is also taken into consideration with respect to shape and physical state (amorphous/crystal), as it may cause irritation in the ocular tissue [27,28]. Thus, keeping optimum particle size in suspensions improves their therapeutic efficacy. Eye drop suspensions in the market, such as TobraDex^®^ suspension, are widely used for bacterial eye infections. The preparation consists of 0.3% tobramycin and 0.1% dexamethasone (Dex). The limitation of this commercial preparation is its high viscosity. Apart from these, adequate excipients (inactive) in eye drop suspensions used as performance enhancers such as suspending agents prevent easy settling or caking, stabilizing/wetting/complexing agents for solubilizing hydrophobic drugs, and the preservatives used as antimicrobial agents are major concerns to be taken into consideration. The redispersing of the suspension drug particle in the container has to be uniform, and an effective dose should be delivered uniformly under therapeutic conditions. The suspension manufacturing techniques are indeed considered, and the fabrication of such dosage form requires suspension aseptic ball milling; thereafter, the preparation is aseptically transferred into a hermetically sealed sterile container [28].

Recently, Scoperet al., sought to minimize the high consistency of TobraDex^®^ and ameliorate absorption of the drug with enhanced bactericidal properties. They modified the preparation as TobraDex ST^®^, with a reduced concentration of Dex to 0.05%. The sedimentation rate of the modified formulation showed very low particle settling over 24 h (3%), compared with marketed TobraDex^®^ (66%). An animal study exhibited high ocular penetration, distribution, and concentration of Dex and tobramycin when treated with TobraDex ST^®^, compared with TobraDex^®^. Thus, the modified suspension preparation was promisingly efficacious, compared with TobraDex^®^, against *S. aureus* and *P. aeruginosa*. The clinical trial of the modified formulation in human subjects also reported a high concentration of tobramycin and Dex in the eye fluid, compared with TobraDex^®^ [29]. 

#### 4.2.3. Solutions

Aqueous solutions for ophthalmic applications must be sterile and are primarily used for rinsing and cleaning the eyeballs. Ophthalmic solutions are safe, easy to use, and noninvasive, and they show rapid action to the local ocular tissues. Aqueous solutions may have some excipients for regulating osmotic pressure and viscosity, as well as simulating the lachrymal fluid and pH. The multidose container may require preservatives [30]. The ophthalmic solution is generally available as eye drop preparation. The solution as eye drop provides immediate drug permeation after instillation, and thereafter, drug concentration declines swiftly. To improve the drug retention time, absorption, and bioavailability in the ocular tissue, compared with eye drop solutions, different additives may be incorporated to enhance viscosity and permeation. For example, cellulose derivatives, such as sodium carboxymethyl cellulose, are generally used as viscosity enhancers. Cyclodextrin is used as a carrier for formulating lipophilic drugs in aqueous solutions and helps in drug release to the biological surfaces [31].

#### 4.2.4. Ointments

The ointment is meant for ocular applications, having a mixture of mineral oil, petrolatum, and paraffin, a solid hydrocarbon that melts at the physiological temperature of the eye (34 °C). Apart from bearing the consistency and biocompatibility of the selected hydrocarbon base, it also offers the advantages of improved contact time and sustained drug release. The ointment preparation may be monophasic or biphasic system due to the presence of water and oil phases. The challenges with the use of ointments as the base are patient related; they may cause soreness and blur vision ascribed to different refractive indices of the lachrymal fluid and ointment base and are prone to inaccurate dosing, and therefore, the wide application of medicated ophthalmic ointment is limited [32].

## 5. Novel Ocular Therapeutic Systems

**Figure 4:**
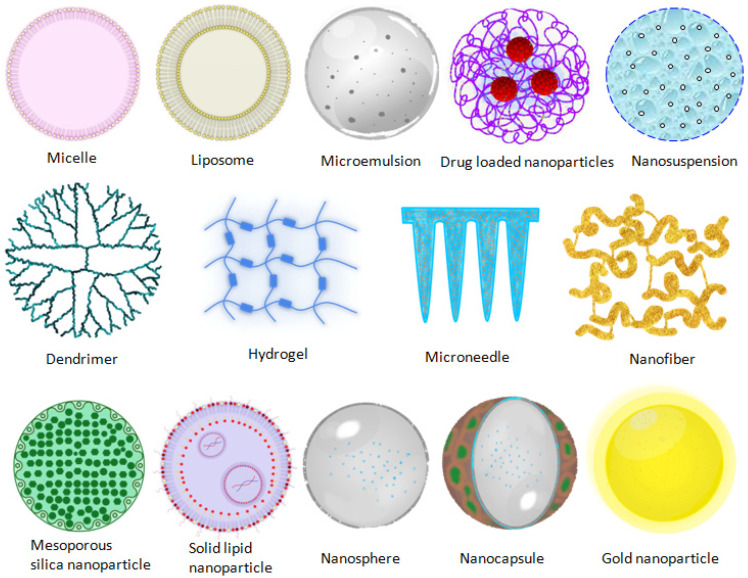
Nanocarriers employed in ocular therapeutic systems.

A few decades ago, many strategies were designed in terms of the therapeutic modality of ocular diseases. The advent of nanotechnology-based therapeutic systems has acquainted the novel facet toward the optimized nanosize particle, which enables minimizing irritation, addressing the poor bioavailability, and improving ocular biocompatibility of therapeutics. The various nanodrug delivery carrier systems employed in the ocular therapeutic system are presented in Figure 4. The nanocarriers enlisted below have shown promised results for enriching the ocular availability of therapeutics. The summarized nanotechnology-based novel drug carrier system is favorable in the treatment of ocular disorders, which is shown in Table 1.

### 5.1. Microemulsion

**Figure 5:**
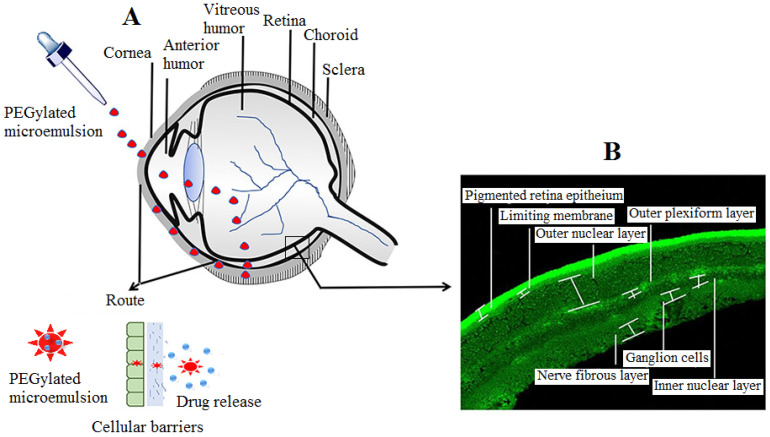
Diagram showing the route of PEGylated ME entry into the posterior segment of eye (**A**) and different parts of retina of eye (**B**). To overcome the cellular barriers, topical PEGylated ME may cross the membrane barriers viz. cornea, conjunctiva, and sclera, thereby preventing opsonization and improving circulation in lachrymal fluid and vitreous humor. Modified from ref. [2]; permission granted from ACS publishers (https://pubs.acs.org/doi/10.1021/acsomega.9b04244, (accessed on 20 December 2021)).

MEs comprise of oil, surfactant, and cosurfactant with medicaments. In general, the thermodynamic stability of oil in water emulsion (o/w) is a consequence of interfacial film around water droplets [70]. ME as a drug delivery vehicle for eye preparation has been widely explored to overcome the various barriers in ocular drug delivery. To improve the significant concentration of drug in the posterior chamber of the eye, an overwhelming study led by Nayak and Misra investigated the PEGylated ME delivery into the posterior segment of the eye (Figure 5). They developed TA-loaded ME, post evaluation in vitro for solubility, emulsion capability, and they construed a pseudoternary phase diagram. The optimum formulation comprised a ratio of oil (Capmul MCM C_8_): surfactant (AccononMC_8-2_): cosurfactant (Transcutol): water of 5:35.5:4.5:55. The emulsion was PEGylated using 1, 2-distearoylphosphatylethanolamine-polyethyleneglycol 2000 (DSPE-PEG 2000). Moreover, the PEGylated drug-loaded ME was characterized and investigated for topical application. The prepared PEGylated ME was observed to be stable, homogenous, and nonirritant to the eye, after animal study, and had the potential to achieve the target to the posterior segment of the eye after topical administration [2].

**Figure 6:**
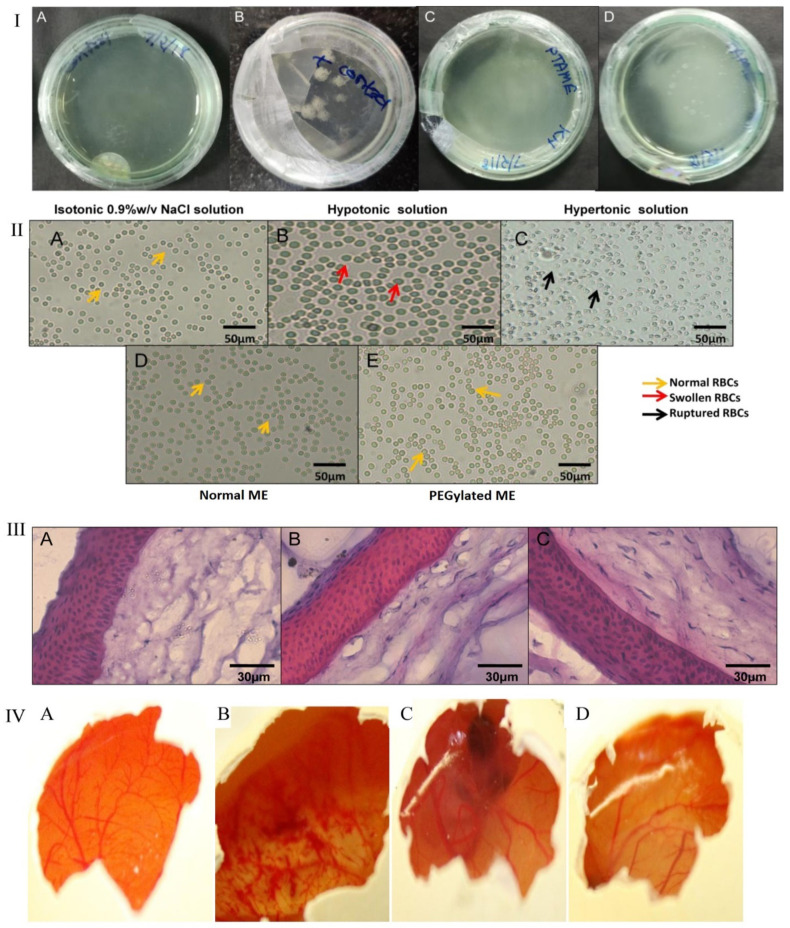
(**I**) Culture plate’s sterility test on incubation with (**A**) saline solution, (**B**) positive control, (**C**) PEGylated ME, and (**D**) normal ME; (**II**) tonicity evaluation, RBCs treated with (**A**) saline solution, (**B**) hypotonic solution, (**C**) hypertonic solution, (**D**) normal ME, and (**E**) microscopy of PEGylated ME; (**III**) hematoxylin-and-eosin-stained corneal sections treated with (**A**) saline solution, (**B**) normal ME, and (**C**) PEGylated ME observed under a microscope; (**IV**) corneal hydration test. Images captured after 3 h of hen’s egg membrane treated with (**A**) saline solution, (**B**) NaOH solution, (**C**) normal ME, and (**D**) PEGylated ME. Modified from ref. [2] (https://pubs.acs.org/doi/10.1021/acsomega.9b04244, (accessed on 20 December 2021)).

The findings shown in Figure 6 illustrate that aseptically developed ME was free of microbial contamination and did not corroborate the microbial growth. In the isotonicity test shown in Figure 6II, the architecture of RBC is maintained in both normal ME and PEGylated ME preparation, which confirmed that both formulations were isotonic to ocular fluid, while they were ruptured in hypertonic (B) and swollen in hypotonic solution (C). The blood and tear fluids possess the same osmolarity, and thus, RBC was utilized for the isotonicity test. Figure 6III shows the nuclei of cornea incubated with normal ME and PEGylated ME were safe, with no risk of side effects using them. Figure 6IV shows the corneal hydration and staining test, which confirmed the nonirritant nature of formulations [2].

Kalam et al., (2016) prepared and evaluated gatifloxacin efficacy in the anterior segment of the eye with respect to good corneal adherence and permeation of the drug and compared it with a conventional eye drop. The prepared ME used an oily phase as isopropyl myristate and a nonionic surfactant such as Tween 80, and Transcutol-P was used as cosurfactant while applying an aqueous titration technique. The formulation appeared to have uniform droplets, and size ranges varied from 51 nm to 74 nm, with a surface charge on ME recorded as 15 mV to 24 mV and optimum physicochemical features desirable for topical instillation. The optimized formulation improved stability and contact time, and resulted in a twofold improvement in bioavailability of the drug, compared with the conventional eye drop. Thus, the ME showed improved intraocular permeation and trans-corneal penetration, as well as preventing precorneal loss and improving absorption of gatifloxacin in the anterior segment of the eye [71]. 

Perminaite et al., developed a novel royal jelly containing 10-hydroxy-2-decenoic acid-based ME for ophthalmic delivery. Royal jelly obtained from natural worker honeybees has potential biological activities such as anti-inflammatory and antioxidant activities. The royal jelly ME prepared by oil titration method comprised royal jelly, Tween 80 as surfactant and Tween 20 as cosurfactant, an oily phase as isopropyl myristate, and water, characterized in vitro. Further, the ME was assessed for irritation in the rabbit’s corneal cell culture. The results demonstrated that ME droplet size was 67.88–124.2 nm, with a polydispersity index of <0.180. The10-hydroxy-2-decenoic acid release depended on the surfactant and cosurfactant ratio employed in the formulation. The cell culture test results indicated that ME was nonirritating [72].

### 5.2. Polymer Micelles

**Figure 7:**
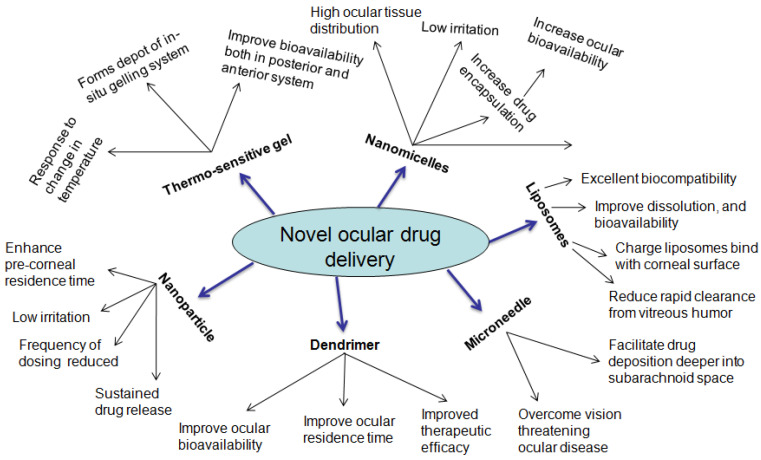
Novel therapeutic strategy in ocular drug delivery.

Nanomicelles are the amphiphilic self-assembling architecture of colloidal particles, with sizes varying from 10 nm to 100 nm, and consist of a hydrophilic head and hydrophobic shell. The nanomicellar delivery system is commonly used to dispense therapeutics into a transparent aqueous solution. It is a widely employed pharmaceutical vehicle for solubilizing hydrophobic drugs. The poor solubility of the drug is a limiting factor for formulating the ocular preparation because of subtherapeutic effects in ocular tissues, which comprises an amphiphilic surfactant or a polymer in the aqueous phase. The carrier system’s important key attributes include easy preparation techniques, high entrapment of the drug, loading, nanosize, and capability to encapsulate hydrophobic drugs and remain in their hydrophobic shell. The carrier system enabled protection against drug degradation and increased drug stability in the aqueous phase. Micelles can be used for the delivery of prodrug, drug–polymer conjugate, and polymer film for sustained release in the ocular system [73]. An illustrative novel therapeutic strategy in ocular drug delivery is shown in Figure 7.

Civiale et al., prepared Dex nanomicelles using copolymers of polyhydroxyethylaspartamide PHEAC (16) and pegylated PHEAC (16) for topical anterior segment drug delivery. The animal studies of Dex nanomicelles were performed in rabbit’s aqueous humor, and results depicted that drug-loaded micelles increased ocular availability of drug by 40%, compared with a Dex suspension. In an attempt to enrich the drug concentration in the posterior ocular tissue, a combination drug (Dex, voclosporin, and rapamycin) with mixed nanomicellar preparation (0.1% and 0.2%) was designed. The tissue distribution analysis after single drop administration indicated that nanomicellar preparation containing multidrug enabled therapeutic concentrations to be achieved in the posterior chamber of the eye. These findings suggest that nanosize micelle could evade the physiological barriers in the ocular region and efficiently deliver drug carriers to the posterior ocular tissues [33]. 

Junnuthula et al., developed self-assembled block-copolymers of polymeric micelles and polymersomes and investigated for physicochemical features, including interactions and retention with vitreous liquid. Furthermore, they performed in vivo study in rabbits for ocular kinetics via intravitreal injections. Their findings revealed that polymersomes retention prolonged in the ocular tissue and deposited more to the retina, as well as the optic nerve, in the head region [73].

Alami-Milani et al., designed Dex-encapsulated polycaprolactone–polyethylene glycol–polycaprolactone micelles, and an ex vivo permeation test indicated thatmicelles could potentially deliver the hydrophobic Dex in the ocular tissue [74]. Vaishya et al., (2014) developed and characterized Dex-loaded polymeric nanomicelles for the posterior segment uveitis treatment. Pertaining to this, a low-molecular-weight di-block copolymer was synthesized and evaluated in vitro for the formulation of critical micelle concentration and tested in ocular cells for toxicity. The nanomicelles size incurred 25–30 nm, with uniform distribution and a polydispersity index of 0.125. The permeation of drug-loaded nanomicelles was raised by 2-times across the conjunctival cell line and by 2.5-times across the excised rabbit sclera, compared with a drug suspension. Thus, nanomicellar preparation herein developed could achieve therapeutic levels in the posterior region of the eye after topical instillation [75]. 

The topical treatment of posterior uveitis is noteworthy but goes against conventional therapy to achieve therapeutic concentration. In a similar attempt, to maximize the drug concentration in the posterior eye segment, Nikita et al., designed an everolimus-loaded nanomicellar preparation using Soluplus^®^, a grafted copolymer of polyvinyl caprolactam–polyvinyl alcohol–polyethylene glycol (PVCL–PVA–PEG) for enhanced permeation and bioavailability in the ocular epithelia to treat the ocular uveitis. The nanomicelles had a size of 65.55 nm and low CMC (7.2 µg/mL). The surface analysis was found to be uniform, spherical, and smooth. The drug entrapment was high, and the release profile was sustained, compared with adrug suspension. The permeation study in the cornea of goat mucosa suggested higher permeation across the cornea than drug suspension. Further, the higher drug permeation of nanomicelles was confirmed by confocal microscopy. Overall, the outcomes of the study clearly pointed to higher drug access and enhanced bioavailability of everolimus-loaded nanomicelles and revealed that these nanocarriers could be promisingly employed in the treatment of ocular uveitis [76].

Further, Patel et al. prepared Dex-loaded nanomicelles of polyoxyl 40 stearate and polysorbate 80 and characterized in vitro for solubility, critical micellar concentration formation, micelles size, zeta potential, surface morphology, drug release, and efficacy in an animal model. The ocular drug tolerance and drug distribution in ocular tissue were investigated in an animal model after single and multiple dosing. The developed nanomicelles containing Dex (0.1% *w*/*v*) showed micelles size of 13.3 ± 0.4 (placebo) and 14.5 ± 0.4 nm (drug-loaded nanomicelles). The TEM image observed a spherical, stable, and uniform micelle size. The animal testing revealed no inflammation, redness, or irritation when compared with control. The drug concentration was sufficient to exert a therapeutic effect in the cul-de-sac cavity after topical use in the rabbit’s eye. The generated novel nanomicelles enabled the solubilization of 0.1% Dex hydrophobic core and potentially delivered the drug in the posterior segment of the eye for treatment of posterior uveitis [77].

For active targeting based on peptide transporter-1, Xu et al. designed nanomicellesfor ocular delivery. They prepared chitosan oligosaccharide–valylvaline–stearic acid (CSO–VV–SA) nanomicelles and castor oil-40/octoxynol-40 (HCO-40/OC-40) mixed nanomicelles. The in vitro cytotoxicity assay produced no significant difference in human corneal epithelial cells (HCEpiCs) and conjunctival epithelial cells (HConEpiCs). The inhibitory test confirmed the active transport of CSO–VV–SA nanomicelles through the chosen transporter. The fluorescence study confirmed the active transport of CSO–VV–SA nanomicelles by PepT-1 in the posterior segment via the conjunctiva. The animal study demonstrated precorneal retention of Dex from both nanomicelles of more than 3 h. The results indicated that CSO–VV–SA nanomicelles could be novel carriers, with promising testing potential in clinical applications [78]. 

### 5.3. Nanoparticles

NPs are colloidal nanoparticulate carriers whose size generally varies in the range of 10–1000 nm. For ocular drug delivery, NPs comprise a mixture of protein, lipids, and or polymers derived from synthetics such as PLGA, polylactic acid (PLA), albumin, alginic acid, chitosan–alginate, and polycaprolactone. NPs improved the ocular passage of hydrophobic drugs by disrupting superficial ocular barriers and granting systemic access to medicaments from specific sites [79]. The drug-encapsulated NPs have desirable biological characteristics related to the enhanced ocular residence time from the dosage form, reduced toxicity, and increased drug penetration capability deeper to the ocular tissue, and concomitant with reduced drug loss from the precorneal spaces due to rapid tear fluid turnover [80]. NPs are promising carriers of drug candidates in ocular delivery, owing to their small scale, little eye irritation, and prolonged drug release, hence the reduction in dosing frequency. Due to easy elimination from the precorneal pocket, as seen generally with aqueous drug solutions, NPs administration is designed with a mucoadhesive feature likely to aid for more time in the precorneal chamber. For this, chitosan polyethylene glycol (PEG) and hyaluronic acid are preferably used to increase the pre-corneal residence time of drug-loaded NPs [81]. 

NPs with chitosan polymer are widely researched for ameliorating drug concentrations in the precorneal cavity by improving the ocular residence time. The chitosan is positively charged, which binds effectively with the negatively charged surface of the cornea and thus improves corneal residence time and minimizes the precorneal clearance. It was illustrated that natamycin chitosan/lecithin NPs improved ocular bioavailability by 1.47 fold and reduced precorneal clearance by 7.40 fold, at a low dose, and reduced frequency of instillation in rabbit’s eye, compared with a marketed suspension [82]. Musumeci et al., reported that melatonin-encapsulated PLGA–PEG NPs were effective in lowering intraocular pressure (IOP), compared with melatonin PLGA NPs and drug solution of an equivalent concentration in the rabbit’s eye. It was indicated that the decreased surface charge of PLGA–PEG, compared with PLGA alone, well accorded and enhanced interaction of NPs with eye surface, resulting in better hypotensive outcomes for a prolonged time [34].

Zhang et al., instilled Dex in rabbit’s eye through intravitreal injection and investigated pharmacokinetics and ocular tolerance of the drug from PLGA NPs. The results concluded that DEX-encapsulated NPs showed sustained release of the drug for50 days. The vitreous humor reported constant drug levels (3.85 mg/L) for 30 days. The results implied that Dex NPs via intravitreal injection provided sustained release in the posterior segment of the eye [35]. Chi et al., studied hybrid NPs and nanosheets for enhanced cellular uptake in the ocular tissues, using peptide transporter-1. They prepared and characterized nanocarriers in vitro. Both nanosheets and hybrid NPs indicated the sustained type of drug release in vitro and enhanced the precorneal retention in vivo, but hybrid NPs showed higher permeability in vitro than nanosheets. Furthermore, a cellular uptake study on HCEpiCs and ARPE-19 cells showed endocytosis based on actively transported PepT-1 and higher drug internalization, both from hybrid NPs and nanosheets. Thus, it was concluded that hybrid NPs are promising carriers for ophthalmic instillation in the mid-posterior region, whereas nanosheets are ideal for ocular diseases [36]. 

Yu et al., developed several Dex–glycol chitosan (Dex–GCS) conjugate by chemical synthesis and characterized for UV–Visible spectroscopy, infrared spectroscopy, and X-ray diffraction technology. The conjugate self-assembled into NPs with a size range of 277–289 nm and a positive surface charge of +15 mV. The particles were ascertained as spherical via transmission electron microscopy (TEM). Moreover, mucoadhesive properties of Dex-GCS NPs with varying concentrations of mucin were evaluated in vitro. Dex release in phosphate-buffered saline (PBS, pH = 7.4) expressed progressive drug release till 8 h and then reached plateau upto 48 h. The cytotoxicity against L929, HCEC, and RAW 264.7 cells of the formulation was tested after incubation of 24 h and showed similar efficacy to Dex sodium phosphate (Dexp) in lipopolysaccharide (LPS)-activated RAW 264.7 macrophages. More interestingly, the developed Dex-GCS NPs established effective ocular tolerance and precorneal retention, compared with an aqueous preparation, indicating that the self-assembled Dex-GCS NPs appear to be an anticipated system for ocular therapeutic delivery [83].

### 5.4. Nanoparticulate Targeting in Retinoblastoma (RB)

Retinoblastoma is encountered during childhood, and the incidence rate is more prevalent in children under the age of 5 years. The survival rate of the cancer is high but may lead to severe complications such as vision loss and even death if not diagnosed and treated timely [84]. After detection of cancer, medical intervention relates to chemotherapy, radiotherapy, and or surgery, which are meant to improve patient survival. The NP-based drug delivery investigated in RB led to improved drug delivery in the posterior eye segment and also increased the intravitreal half-life (t_1/2_) of chemotherapeutic agents with potential outcomes in retinal cancer [85]. A nanoparticle targeting based on rationale design essentially incorporates functionalized moieties or ligands for potential cellular uptake, and cellular internalization of therapeutics has been reported in several publications [86,87,88,89]. Several types of conjugating agents—namely, epithelial growth factor receptor (EGFR), folic acid, transferrin, cell penetration peptide, and proteins, are used for surface functionalization processes, depending upon the dominancy of specific receptor to the target site [90]. Further, it has been observed that the surface alteration of nanocarrier using polyethylene glycol (PEG) improved the NP uptake due to colloidal stability, reduced protein adsorption, and less opsonization, thus improving the intravitreal transport to target cells [91]. Sims et al. designed functionalized melphalan-loaded poly(lactic-co-glycolic acid) (PLGA) NPs to increase the intravitreal drug delivery through positive cell association and improved efficacy in retinoblastoma cells. They compared the cell association potential and efficacy in retinoblastoma cells surface-modified PLGA NPs with unmodified NPs. They observed prominent cell association, cell internalization, and enhanced efficacy with surface functionalized MPG-NPs after 24 h of administration, compared with unmodified NPs. In another study, topotecan-bearing mesoporous silica NPs with folate conjugation had enhanced drug efficacy in RB treatment. The nanosized particles demonstrated sustained drug release and superiorcell uptake in Y79 RB cells, compared with nontargeted NPs [92].

### 5.5. Nanosuspensions

Using conventional techniques to formulate hydrophobic substances is highly challenging. The application of nanotechnology in formulating hydrophobic drug substances such as nanosuspension is desirable to address the problem associated with drug molecules. Nanosuspension is a colloidal carrier of dispersed drug substances of submicron particle size, which are, in turn, stabilized by formulation additives such as surfactants or polymers. In ocular drug delivery, the method offers many benefits such as ease of sterilization in formulating the eye drop, minimizing ocular irritation, improving precorneal residence, as well as enhancing ocular drug absorption. Many studies well reported the improved glucocorticoids absorption via ocular drug delivery. Glucocorticoids such as Dex, prednisolone, and hydrocortisone have been widely suggested for the therapeutic modality of inflammatory conditions in ocular tissues of the anterior segment. However, the conventionally established treatment using these drugs needs multiple dosing, resulting in large cumulative doses that may further lead to complicationssuch as cataracts, optic nerve damage, or glaucoma. Kassem et al. successfully developed glucocorticoids (Dex, prednisolone, and hydrocortisone) nanosuspension, and the formulation was effective in reducing the intraocular pressure in rabbit’s eye [37]. 

Recently, Yan et al., compared mucus-penetrating particles (MPPs) and cationic NPs suspension having cyclosporine A (CsA) in terms of ocular bioavailability. This study further clarified the mucous permeation capacity of MPPs and mucous-retaining capability of cationic NPs, although both preparations were capable of prolonging the ocular residence time of the drug on the eye surface. Both cationic nanosuspensions and MPP nanosuspensions (drug core) were prepared by applying an antisolvent precipitation technique. The X-ray analysis revealed that CsA was in an amorphous state in both formulations. The in vitro mucoadhesion analysis showed that cationic nanosuspensions interacted 5.0–6.0 times higher with pig mucin, compared with MPP nanosuspensions (drug core). The permeation study on drug-core MPP nanosuspensions indicated that apparent permeability (Papp) value was 5.0–10.0 times greater than cationic nanosuspensions. The in vivo ocular bioavailability showed CsA concentration in cationic and MPP (drug-core) nanosuspensions were 13,641.10 ng/g and 11,436.07 ng/g, respectively, which was significantly greater than conventional nanosuspension (8310.762 ng/g). These results indicated that both cationic and MPP nanosuspensions were effective in delivering the CsA concentration (10–20 μg/g) to the anterior chamber using eye drops. Therefore, cationic nanosuspensions look promising, as they provided more ocular bioavailability than MPP nanosuspensions [93].

Boddeda et al., prepared flurbiprofen (FB)-encapsulated polymeric nanosuspension for enriching the bioavailability in the ocular region. The nanosuspension was developed by a solvent displacement technique while optimizing the process variables—namely, drug and polymer ratios and aqueous-to-non aqueous solvent ratio, as well as their impact on formulation characteristics including size, drug release, and ocular tolerance. The developed nanosuspension showed a spherical particle shape, a particle diameter of around 100 nm to 200 nm, with surface charge ranging from +6.6 ± 2.2 to +19.0 ± 3.1 mV, and drug encapsulation was recorded between 54.67 ± 3.4 and 90.32 ± 3.2%. The nanosuspension underwent sustained release of drug (60%) over 12 h, compared with a marketed preparation (Flur eye drops). In vivo study of an animal model reported that it was nonirritant and safe, based on histopathological studies. The FB-loaded Eudragit nanosuspension proved to be safe, stable, and suitable for ocular use [38].

### 5.6. Liposomes

Liposomes are widely sought drug delivery carriers with vast applications in different areas of biomedical sciences, including topical applications. They are lipid-based spherical vesicles that have one or more cell membranes such as phospholipid bilayers encasing the aqueous phase and proved as promising drug delivery carriers for ocular disease therapy due to the enhanced ocular residence time for drug absorption. The size of the vesicle ranges between 10 nm and 1000 nm, depending upon the phospholipid layer:unilamellar vesicles (10–100 nm), bilamellar vesicles (100–300 nm), and multilamellar vesicles (>300 nm). In recent times, liposomal drug carrier remains a point of interest for ocular drug delivery. The liposome is an ideal drug carrier owing to its remarkable biocompatibility, high degradability, flexibility, nonimmunogenicity, nontoxicity, and being mimetic to cell membrane architect, enabling the encapsulation of both lipophilic and hydrophilic drugs and delivering the medicaments effectively in both anterior and posterior chambers [94]. Various studies investigated the liposomal drug delivery for improving dissolution, bioavailability, precorneal penetration, increased residence time, and targeted action [39,95,96]. 

**Figure 8:**
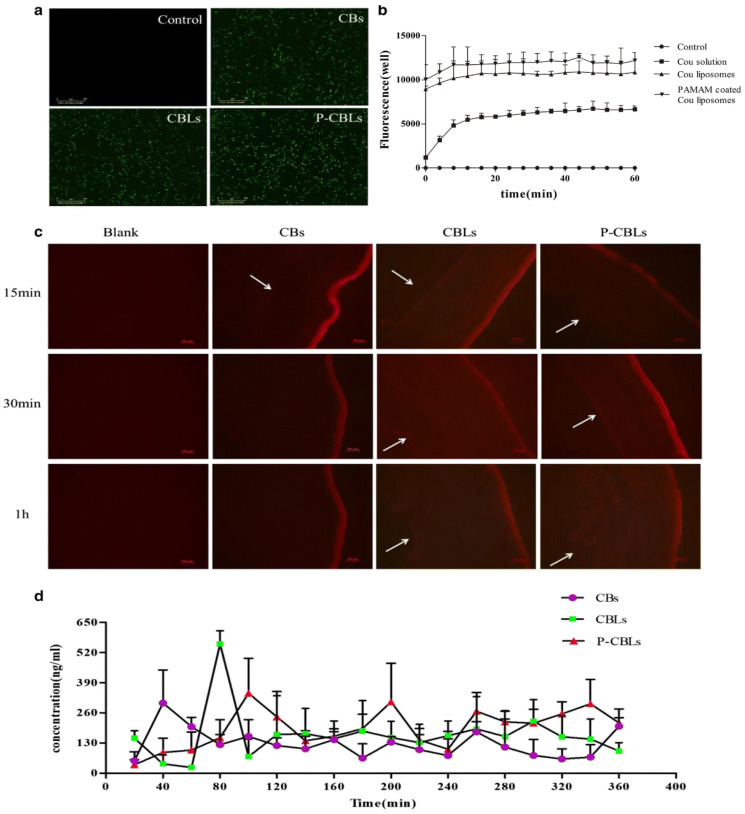
Investigation of liposomal effectiveness in ocular drug delivery: (**a**) the fluorescence images of different formulations with Coumarin (Cou) using cell analyzer. The cellular uptake after 24 h of human corneal epithelial cells (HCECs) (scale bar = 300 μm); (**b**) formulations intake count; (**c**) the Nile red-stained formulation distribution images captured in cornea; the corneal endothelium indicated by arrow (scale bar = 50 μm); (**d**) in vivo pharmacokinetic parameters after topical instillation of different formulations. Permission under Commons Attribution 4.0 International License [95]. (http://creativecommons.org/licenses/by/4.0/, (accessed on 15 November 2021)).

Age-related macular degeneration (AMD) in the eye is a leading problem associated with the central region of the retina, i.e., the macula of the eye, which may lead to visual deficiency and at later stages to blindness. To improve the solubility and bioavailability of berberine hydrochloride (BBH) and chrysophanol (CHR) for the treatment of ocular diseases based on active biological response related to anti-inflammatory, antioxidative, and antiangiogenic effects, Lai et al. developed PAMAM-coated liposomes. The PAMAM-coated liposomes indicated considerable cellular permeability in the corneal cells and increased bioadhesion on the corneal epithelium of the rabbit model. The coated liposomes improved drug absorption and acted apparently as protection for the retinal pigment cells and also protected the rat’s retina after photooxidative injury. The formulation of liposome pointed to no side effects post investigation of ocular morphology in the rabbit. The cellular internalization of the developed formulation was investigated in HCEC cells after incubation of 24 h. As indicated in Figure 8a,b, PAMAM-coated coumarin (Cou) liposome showed stronger fluorescence intensity, compared with normal liposomal formulations, after 1 h of topical administration. The study suggests that PAMAM-coated Cou liposomes may significantly elicit the cell uptake of therapeutics from carrier systems, compared with normal liposomes [95].

The different preparations—namely, chrysophanol–berberine hydrochloride suspensions (CBs), compound liposomes (CBLs), and PAMAM-coated compound liposomes (P-CBLs) were used to examine the transcorneal permeability. Each of the formulations can similarly penetrate the corneal epithelium, as indicated by the fluorescence intensity for 15 min initially after topical instillation. As regards moving time, the concentration of CBLs and P-CBLs were more detected in the corneal epithelium, shown by high fluorescence intensity. High drug retention in the ocular tissue was also confirmed because of the lack of drug in tear fluid. Moreover, for CBs, the fluorescence intensity was diminished in the corneal endothelium, indicating that they are unable to permeate through the corneal epithelium (Figure 8c). A pharmacokinetic study was performed using formulation CBs, CBLs, and P-CBLs in the rabbit’s eye. Notably, the outcome of the study revealed that C_max_ of BBH in the aqueous humor with instillation of P-CBLs and CBLs were 1.719 and 1.23-times greater than CBs. The bioactivity of BBH loaded liposomes was 1.33 times greater than BBH-Loaded CBs, whereas P-CBLs raised the bioactivity by 1.6343 times vis à vis CBs. Therefore, the PAMAM-coated liposomal system showed potential utility in treating complex ocular ailments [95].

**Figure 9:**
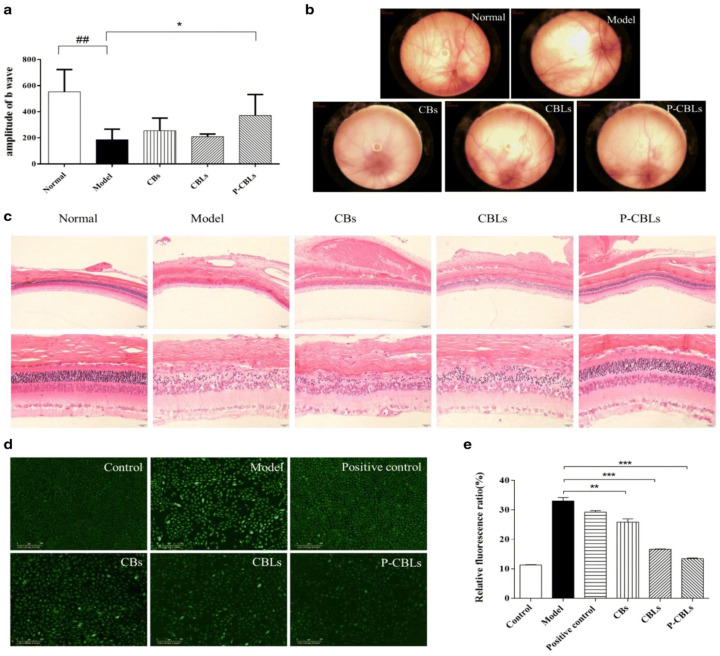
Effective drug delivery of liposomes into posterior ocular segment. Protection against photo-oxidative retinal damage: (**a**) the b-wave amplitude alteration following topical administration of different formulations for up to 14days; (**b**) retinographic images of various formulations treatment; (**c**) the protection efficacy of various hematoxylin-and-eosin (H and E)-stained formulations in the retina (scale bar = 20 μm); (**d**) images of in vitro anti-ROS efficacy taken with a long-term real-time dynamic live cell imaging analyzer: (**e**) ROS levels of various formulations. Data are expressed as mean ± SD (*n* = 3). * *p * < 0.05, ## *p * < 0.01, ** *p * < 0.01, *** *p * < 0.001. Permission under Commons Attribution 4.0 International License [95]. (http://creativecommons.org/licenses/by/4.0/, (accessed on 15 November 2021)).

Moreover, pharmacodynamic studies were conducted to investigate the therapeutic efficacy in the ocular region, with liposome formulations, in light-induced retinal tissue damaged rat’s model. The pentobarbital sodium (3%) was injected viathe intraperitoneal routeinto different groups of animals—normal saline, placebo liposome, CB, CBL, and P-CBL groups. Among these formulations, P-CBLs induced the highest protection in the reversal of retinal function in photo-exposed rats. Flash electroretinogram after 14 days by light damage of retinal tissue indicated a significant increase in b-wave responses in P-CBLs-treated rats, compared with other formulation groups, as shown in (Figure 9a). The normal-saline-group-treated rats kept their intact retinal vessels, and the background of the fundus was clearly seen. The rat vessels treated with the blank liposome group showed some severe manifestation in the fundus. The P-CBLs had no impact on the retinal blood vessels, but they improved the reflection area in the eye, as shown in (Figure 9b). Compared with the normal saline group, the blank liposome group decreased the numbers of outer nuclear layer cells and led to the thinning of the layer significantly. Adversely, histopathological examination of the eye section showed evidence of protective impression in the retina in P-CBL-instilled rats after ocular cell injury caused by the photo-oxidative process. The morphological analysis revealed clear layers of retinal structure and well-stained nuclear layers in P-CBL-treated rats (Figure 9c). The antioxidant assay showed P-CBLs were highly potent in reducing ROS levels as per the relative fluorescence ratio, compared with CHR and BBH, as shown in Figure 9d,e [95].

**Figure 10:**
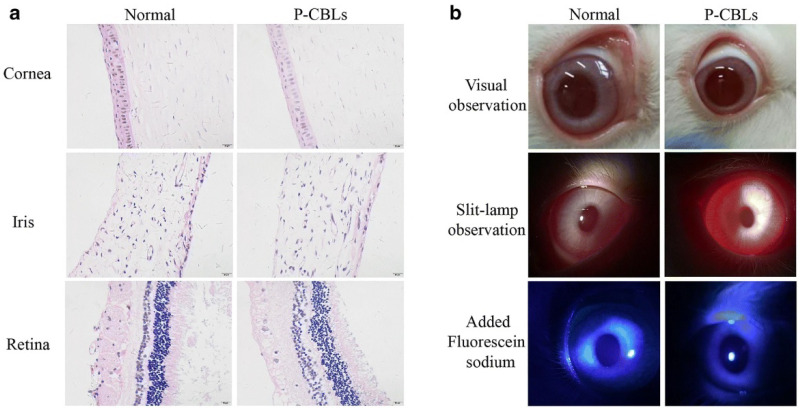
(**a**,**b**) Ocular irritation studies: (**a**) typical histological image of formulation, P-CBLs after instillation for 14 successive days (scale bar = 20 μm); (**b**) ocular surface examination using a silt lamp and camera, post staining with 0.5 % sodium fluorescein. The ocular irritation studies manifested no injuries or abnormalities in either part of the cornea, conjunctiva, or iris of the eye (**a**). The 0.5% sodium fluorescein stained ocular surface observed under a silt lamp and camera found no edema or injuries, which further substantiated the safe and protective nature of P-CBLs (**b**). Permission under Commons Attribution 4.0 International License [95]. (http://creativecommons.org/licenses/by/4.0/, (accessed on 15 November 2021)).

Moreover, the ocular irritation study in rabbits was performed using the same formulation with equivalent drug doses such as CBs, CBLs, and P-CBLs, and the ocular surface was analyzed using the Draize eye test. The tissue histology analysis revealed that the cornea, iris, and conjunctiva were safe, and no tissue damage was seen in thegroup after 14 days of instillation of P-CBLs (Figure 10a). The stained ocular surface with 0.5% sodium fluorescein observed under a silt lamp and camera showed no edema or injuries, demonstrating the safe and protective nature of P-CBLs (Figure 10b) [95].

A recent study led by Natarajan et al., investigated a latanoprost liposomal preparation for delivery to the anterior segment of the ocular tissues. The instillation of single liposomal formulation via subconjunctival injection in rabbit’s eye brought forth sustained lowering effect of IOP for upto 50 days, which is comparable to the conventional eye drop formulation. Cationic liposomes experienced better drug delivery efficacy in the posterior ocular segment, compared with anionic or neutral liposomes, by using positively charged lipid or mucoadhesive, there by improving ocular residence time of the drug. For instance, stearylamine and didodecyldimethylammonium bromide are generally employed in designing cationic liposomes [40].

To improve the antibiotic efficacy in topical instillation dos Santos et al., encapsulated besifloxacin into liposomes with additives as positively charged amines and investigated the impact of these charges on the drug diffusion process in two approaches—namely, iontophoresis and passive diffusion. The authors hypothesized that the charge present on the liposome surface could enhance the burst release due to electromigration upon application of electricity and improve the penetration efficiency and residence time of formulation. Herein, liposomes prepared by using phosphatidylcholine (LP PC) or phosphatidylcholine and spermine (LP PC:SPM) were stable, indicating the mucoadhesive property and that they were compatible withthe ocular tissues. Furthermore, electron resonance spectroscopy exhibited that drug and excipients incorporated in liposomal preparation did not interfere with membrane fluidity, structural integrity, and stability in the iontophoretic state. The liposome (LP PC) showed a mean diameter of ~177 nm and zeta potential of −5.7 ± 0.3 mV, and for liposome (LP PC:SPM), the mean diameter and surface charge were ~175 nm and +19.5 ± 1.0 mV, respectively. The minimum inhibitory concentration (MIC) and the minimal bactericide concentration (MBC) of the developed liposomes investigated for *P. aeruginosa* indicated lower MIC and MBC than the marketed preparation (Besivance). Both formulations showed the same efficacy, and surface charge incorporation on liposomes was not beneficial in iontophoretic therapy. On the other hand, as investigated in an ocular model in vitro, passive diffusion/penetration of a drug that simulates the tear fluid remains challenging in passive delivery of the formulation due to ocular resistance to the formulation. The result anticipated that liposomes LP PC:SPM expressed higher drug penetration than the marketed preparation, Besivance. Therefore, besifloxacin-loaded positive (+) liposomes ameliorated the passive delivery of drug upon topical instillation and could be considered a novel approach to enhance the ophthalmic disease therapy [39].

Rajala et al. examined the utility of liposome–protamine–DNA complex (LPD) in gene delivery through a subretinal manner. A biomimetic virus was designed to havemodifications in cellular and signaling peptides for the delivery of retinal pigment epithelium protein 65 (Rpe65) gene for eye disease treatment in mice. Rpe65 is a key enzyme that controls the photochemical 11-cis-retinal and helps to see objects. The results indicated that modified liposome showed effective Rpe65 gene delivery in a specific and further alleviated long-term expression of the Rpe65 gene in Rpe65 knockout mice, resulting inthe rectification of blindness in vivo [41].

In another study, acyclovir encapsulated in positively (+) and negatively (−) chargedliposomes were fabricated using stearylamine (cationic) and dicetylphosphate (anionic) charge-inducing agents. The drug concentration from positively charged liposomes in the cornea of the rabbit’s eyewas higher than the liposomes of negatively charged vesicle and plain acyclovir when administeredviatopical instillation after 2.5 h. The observed concentration drug in the cornea for the drug solution, andpositively and negatively charged liposomes were 253.3 ± 72.0, 1093.3 ± 279.7, and 571.7 ± 105.3 ng/g. The increase in drug absorption from positively charged liposomes was 2-times higher than negatively charged liposomes and 5-times higher than drug solution, indicating that positive surface liposomes havea great affinity with the negatively charged corneal surface, which may be ascribed to electrostatic interaction, thereby increasing the ocular residence time and drug absorption [42].

In the posterior segment, drug delivery of liposomal preparation is to be more concentric on elating the t_1/2_ of the drug by foreshortening the fluid clearance from vitreous humor and protecting degradable molecules viz., peptides and nucleotides, and furnishing sustained release of the drug. Referring to this, liposome enabled the increase influconazole t_1/2_ in the vitreous humor of rabbit eye approximately 8-times higher than plain drug [97].

In a similar study, a tacrolimus liposome was prepared for effective therapy of uveoretinitis. After i.v. administration of tacrolimus-loaded liposome, the concentration of drug in the vitreous humor level heightened to >50 ng/mL and showed sustained release behavior for 14 days. Thus, the tacrolimus liposomal formulation was proved more efficacious in limiting uveoretinitis, compared with the drug solution and also overcome the toxicity caused inside the retinal cells [98]. Among alarge number of liposomal formulations in the preclinical and clinical phases, few of them are commercially available such as Visudyne^®^ and Tears again^®^ used for the treatment of ocular diseases. Visudyne^®^, a liposomal preparation having verteporfin as a photosensitizer, is applied in photodynamic therapy for the growth of new blood vessels in choroidal cellsassociated with macular degeneration (age related), ocular infection, and myopia [99].

### 5.7. Dendrimer

Dendrimers are nanoscale, star-shaped multibranched structures comprising polymeric chains. They are accessible in various molecular grades with terminal end positions of –NH2, OH, and –COOH groups. The end position functional moiety may be used to functionalize with various ligands or targeting moieties. The multibranched dendrimer may allow a large number of lipophilic or hydrophilic drug moieties to become entrapped. Recently, using dendrimer as a carrier for delivering a drug through different routes in the ocular cavity has been reported, with a promising outcome of PAMAM dendrimer. 

Vandamme et al., employed the utility of PAMAM dendrimers as ocular drug delivery vehicles of pilocarpine nitrate and tropicamide delivery in glaucoma miotic and mydriatic activity. The ocular retention time of PAMAM solutions, fluorescein saline, and fluorescein in carbopol solution (0.2% *w*/*v*) was investigated in the rabbit eye. The average ocular residence period of PAMAM and carbopol solution was accounted higher than the normal saline. Thus, dendrimers as ocular vehicles were suggested as desirable alternatives for enriching ophthalmic residence time and improving ocular drug availability, with better outcomes in the ocular therapy. For instance, pilocarpine nitrate- and tropicamide-loaded PAMAM dendrimers showed prominent miotic and mydriatic activity when administered in albino rabbits [43].

To overcome corneal inflammation and reduce dosing frequency, Soibermanet al., explored Dex-loaded hyaluronic acid crosslinked G4-PAMAM dendrimer gel for subconjunctival injection as a potential ocular delivery approach for sustained release and increased absorption of D-Dex. The therapeutic efficacy of the formulation was tested on a rat model. Herein, the fluorescently labeled dendrimers (D-Cy5) loaded in the gel were investigated for D-Cy5 release in vivo. The D-Cy5 was specifically released in the target inflamed tissue and remained confined to the corneal macrophages in the infected rat. Further, improvement in inflamed tissue of the cornea, corneal clarity, and reduced neovascularization by subconjunctival application of D-Dex gels were clinically proven over a period of 2weeksin comparison with the freeDex. The outcomes of the study established that D-Dex dendrimer was more effective in weakening the corneal inflammation than freeDex, probably through inactivating macrophage and cytokines expression (pro-inflammatory). The developed injectable gel of D-Dex could have potential as a treatment of inflammatory disorders in the ocular tissues related to keratitis, dry eye syndrome, as well as postsurgical problems [100].

Several drugs have been investigated in polyamidoamine dendrimers as novel platforms for ophthalmic drug release in the aqueous solution. Herein, the authors developed a fast-dissolving dendrimer-based nanofiber (DNF) based on dendrimer as a vehicle for topical drug delivery of brimonidine tartrate (BT) in glaucoma treatment. The drug release kinetics and safety concerns of the nanofiber were securely investigated both in vitro and in vivo and showed zero toxicity at therapeutic dose in cultured cells and no irritation caused in the normotensive rat model. The intraocular pressure post administration of a single dose having equivalent amounts of the drug in DNF and BT solutions was measured the same. The DNF indicated significant improvement in efficacy, compared with the BT solution, observed for 3weeks after once-daily dosing suggested that dendrimer nanofibers could act as alternatives for effective drug delivery used as eye drops for glaucoma therapy [44].

### 5.8. In Situ Gel

Hydrogel is a crosslinked polymeric system that has wide applications in medical sciences including drug delivery and tissue engineering. In ocular therapy, hydrogels are used as promising carriers for drug delivery in a cul-de-sac cavity on account of biocompatibility and their capability to hold both hydrophilic and lipophilic drug-loaded systems, protecting them for an adequate amount of time. Hydrogel acts as a drug depot, on-demand drug release, and tunable cargo could maintain a therapeutic window, thus enhancing drug absorption [101]. The polymeric structure can hold a large amount of water and or biological fluid in a swollen state. In situ hydrogel is a polymeric solution in an aqueous medium that has phase transition characteristics of sol-gel via physicochemical crosslinking, resulting in the formation of a viscoelastic gel. The gel-forming capacity can be enhanced by alteration in heat or temperature, medium pH, and ions or may be developed through UV irradiation. The stimuli-responsive thermosensitive gel is widely explored for a number of therapeutics in ocular drug delivery [102]. There are several thermo-gelling polymers that have been accounted for in the literature for ocular delivery, including poloxamers, poly (*N*-isopropylacrylamide), copolymers of polycaprolactone, polyester block copolymers polyethylene glycol, poly (lactide), and glycolide, as well as chitosan. The thermogelling polymer at room temperature remains in a liquid state but can solidify into gel post injection at a physiological temperature [103]. 

Phua et al., rationally developed a preparation to outweigh the limitation of poor bioavailability in ophthalmic treatments. The preparation comprised thermosensitive hydrogels of pluronicF-127 meant, to strategically enhance the bioavailability by improving ocular residence of drug-encapsulated nanoliposomes dispersed in thermosensitive hydrogels. They prepared a depot preparation of nanoliposomes for subconjunctival injections. Senicapoc-loaded nanoliposomes showed sustained release from nanoliposome and hydrogel preparations. The in vivo study in Sprague Dawley rats showed a 12-fold increase in ocular residence time, with 24% hydrogel preparation for 1 h, compared with 5 min for free liposomes, observed with fluorescence measurement. A pharmacokinetic study on flushed tears revealed that hydrogels enabled drug retention for a long time, compared with a viscous preparation (1 h), and drug concentration could also be detected in conjunctival tissues within 24 h post injection [45].

Tacrolimus (TAC) is a hydrophobic drug. The marketed formulation of TAC as eye drop causes poor drug retention in precorneal space, low aqueous stability, and pulse kinetic pattern, overall leading to less drug absorption. Sun and Hu developed TAC-loaded SLN in situ gel (TAC-SLNs ISG) for ocular drug delivery. The optimized formulation was characterized for in vitro performances including drug release properties. The pharmacokinetic and pharmacodynamic studies were also performed, to investigate the impact of formulation in comparison with a drug suspension. The probe sonicated particle of TAC-SLNs ISG had a particle size of 122.3 ± 4.3 nm, and the same was changed nonsignificantly in situ gel. The viscosity of the formulation resulted in pseudoplastic flow. The gelation temperature of the developed gel was 32 °C, and a marked rise in viscosity was observed and formed a rigid gel at a higher temperature. In vitro study illustrated the sustained release of the drug from TAC-SLNs ISG. An in vivo pharmacokinetic study showed that eye drops achieved C_max_ 4657.7 ng/mL within 30 min; on the other hand, TAC-SLNs achieved C_max_ 1892.6 ng/mL in 30 min, and TAC-SLNs-ISG had the highest concentration of 2132.3 ng/mL within 2 h. The lower concentration early on from such formulation was probably due to the sustained release effect. The AUC_0–t_ of TAC-SLNs-ISG and TAC eye drops were 590,355.9 and 222,382.5 ng·min/mL, i.e., 2.65-folds higher for TAC-SLNs-ISG than for TAC eye drops. The data clearly point to the superiority of TAC SLNs-ISG, compared with eye drops [46].

In 2020, Noriakiet al., developed tranilast NPs (ophthalmic TL-NPs formulations) for enhanced drug penetration into the ocular tissues. They designed in situgel, integrating TL-NPs with methylcellulose (MC, 0.5–3%), to improve the ocular residence time of the drug. TL-NPs preparation was fabricated using the bead mill method, and the resulting particle size was ~93 nm. An animal study using rats showed that the concentration of drug in the lacrimal fluid was enhanced when the preparation was developed using the MC (0.5–1.5%) concentration. The drug deposition in the cornea and conjunctiva and the anti-inflammatory effects of TL were observed in rats post instillation, with an ophthalmic TL-NP preparation. The optimized formulations of TL-NPs gel with MC (0.5–1.5%) ensured a long residence and improved contact time in the conjunctiva, compared with a formulation using TL-NPs with 3% MC [47].

### 5.9. Nanocapsules and Nanospheres

Depending upon the structural integrity of polymer NP, this is categorized as nanocapsules or nanospheres. Polymeric nanocapsule has been widely examined and interest in its use as a drug delivery carrier has increased in recent years due to unique nanostructure that comprises an outer part of a polymeric shell and inner part as a liquid or solid core [104]. Katzer et al., prepared prednisolone-containing nanocapsules (NCs) by interfacial deposition technique using polycaprolactone or Eudragit^®^ RS100. The prepared NCs were subjected to physicochemical characterization in vitro for particle size distribution using laser-based spectroscopy and size-tracking analysis. Furthermore, cytotoxicity and ocular irritation were determined on the epithelial cell line of the cornea and chorioallantoic membrane of a rabbit model. NCs were reported to have mean sizes between 100 nm and 300 nm, and prednisolone entrapment was 50%. The NCs showed controlled prednisolone biexponential release for upto 5 h. Both formulations were safe in the CAM test due to being nonirritant and showed no cytotoxicity in corneal epithelial cells in the rabbit. The prednisolone nanocapsule was successfully developed for the first time, for application as eye drops in the treatment of eye inflammation [105].

Rebibo et al., (2021) used tacrolimus as a model drug to treat eye inflammation. Bearing in mind the rapid drug expulsion from the carrier system, a critical challenge in ocular drug instillation, they developed tacrolimus-encapsulated nanocapsules (NCs) for ocular instillation. The developed formulation was assessed for stability and efficacy under different experimental conditions. The characterization of the NCs showed the uniform size, and encapsulation efficiency was high, upto 80%. Furthermore, the lyophilized product showed good stability as per ICH guidelines for 18 and 3 months under long-term and accelerated stability conditions. Moreover, drug-loaded NCs did not show any irritation in the rabbit eye post single and multiple-dose schedules. In addition, ex vivo study of drug penetration on the porcine cornea, as well as pharmacokinetics analyses in various eye compartments of the rabbit, showcased the high retention and permeation of drug from NCs into the anterior chamber of the eye, compared with plain drugs present in the base. An animal study in rats also revealed high tacrolimus concentration in the eye. The designed tacrolimus NC system tested on a murine model of keratitis showed a significant reduction in several inflammatory markers, leading to reduced inflammation in the anterior chamber. The outcomes of the study showed that NCs as eye drops provided clinical and histological effectiveness, chiefly in the inflammation of the posterior eye chamber in the murine model and experimental auto-immune uveitis [48].

Bevacizumab has been employed in ocular therapy in age-related macular degeneration (AMD) in many countries, and due to their short biological t_1/2_, multiple intravitreal injections are required. Li et al., prepared PLGA and PEGylated nano- and microspheres using bevacizumab, with an aim to improve the drug retention time in the ocular cavity. The release profile of the developed formulation evaluated in vitro showed bevacizumab release in a sustained fashion over a period of 3 months [106]. 

Robinson et al. prepared and evaluated epidermal growth factor receptor (EGFR), tyrosine kinase inhibitor (TKI), and AG1478-loaded PLGA microspheres and nanospheres meant for intravitreal injection in rats through an optical nerve crush injury model. In vitro characterization of microspheres and nanospheres revealed particle sizes of ~2.6 μm and ~360 nm. After intravitreal injection, the optic nerve regenerated within two weeks. Moreover, the nanospheres were found to be superior to microspheres in regrowth of the optic nerve. The authors came to the conclusion that nanospheres’ intravitreal installation was more efficacious than microspheres due to delivering the therapeutics in the vitreous humor [49,107].

Giannavola et al., formulated acyclovir-loaded PLA nanosphere colloidal suspensions via a nanoprecipitation technique for ophthalmic drug delivery. They investigated the effect of molecular weight and type of polymer, as well as surfactants concentration used in the formulation on in vitro characteristics of nanosphere. The acyclovir-loaded nanospheres were tested in vivo for ocular drug absorption and the results were compared with a free-drug suspension. Moreover, PLA nanospheres were modified with the PEGylation technique to improve retention of formulation in the aqueous humor. The ocular tolerance test of PLA nanospheres was estimated by a modified Draize test. The drug concentration in the aqueous humor was monitored for 6 h to determined absorption and ocular bioavailability from different formulations. The higher molecular weight polymer led to reduced nanosphere size, whereas the PEGylated formulation showed sustained release, improved pharmacokinetics, and well toleration in the eye. The efficacy of PEGylated PLA nanospheres was reported significantly higher than plain PLA nanospheres [50].

### 5.10. Solid Lipid NPs (SLNPs)

This method is an emerging surrogate to colloidal drug delivery having the advantage of incorporating lipid and polymer NPs into a single system. A patent by Muller and Lucks disclosed SLNPs as stable solutions with a solid lipid core that have the potential for encapsulating medicaments stabilized by a layer of surfactant [108]. The SLNs differ from emulsions and liposomal systems in having high-melting lipids. They are free from chronic and acute toxicity, are biocompatible, and could provide a controlled and targeted release to a specific site. The lipid matrix plays an important role in controlling drug release and protecting the drug from degradable enzymes [109,110].

Fungal eye infection caused by fungal keratitis (FK) is a severe pathogenic condition that may lead to ocular morbidity. In this perspective, Natamycin (NAT)-loaded solid lipid NPs (NAT-SLNs), as first-line treatment of FK, has been designed to combat poor corneal permeation and improve residence time, bioavailability, and efficacy in the ocular tissue by Khames et al., The NAT-SLNs were developed by employing the emulsification–ultrasonication technique. The designed experiment was used to optimize the formulation, and the impacts of concentration of chosen factors, i.e., lipid, surfactant, and sonication time, were studied on particle size, surface charge, and drug encapsulation as responses. The optimized formulation was investigated in vitro for drug release, corneal permeation, and antifungal efficacy, as well as cytotoxicity studies. The outcomes of the optimized preparation reported a mean size of 42 nm and a surface charge of 26 mV, and drug entrapment was ~85%. NAT-SLNs expressed sustained drug release upto 10 h. In addition, NAT-SLNs improved corneal permeation, with a steady-state flux of 11.59 × 10^−2^ cm h^−1^ and permeability coefficient of 3.94 mol h^−1^, compared with plain drug, with a flux and permeability coefficient of 7.28 × 10^−2^ cm h^−1^ and 2.48 mol h^−1^, respectively. The antifungal activity revealed the enhanced zone of inhibition, 8 mm and 6 mm against Aspergillus fumigatus (ATCC 1022) and a clinical isolate of Candida albicans, respectively. The minimum inhibitory concentration (MIC) value was reduced to 2.5 times against each strain of fungus. Furthermore, the developed NAT-SLN formulation was nonirritant to the corneal tissue. The formulation NAT-SLNs resulted in extended drug release, enhanced corneal permeation, higher antifungal efficacy, and no toxic effects on the corneal tissues. Therefore, NAT-SLNs are among the anticipated ocular therapeutic systems for the treatment of corneal keratitis [51].

SLNPs have been widely explored as ocular nanocarrier systems to enhance drug absorption and improve the ocular bioavailability of both hydrophilic and lipophilic drugs. One study investigated the future of clarithromycin encapsulated SLNs in improving the permeation and penetration of drugs in topical ocular therapy. The formulation was developed using high-speed magnetic stirring, followed by the sonication technique. Solubility of the drug with different formulating components—namely, lipid former, surfactant (Tween 80), cosurfactant as transcutol P, and stearic acid was studied. The formulation of clarithromycin SLNs was optimized by statistical design and investigated in vitro for particle size, morphology, stability, ocular permeation, irritation, and pharmacokinetics studies. The drug from SLNs indicated ~80% release in 8 h and was complied with the Weibull kinetic release model. The optimized SLNs revealed a higher drug permeation of 30.45 μg/cm^2^/h, compared with the drug solution. The in vivo studies demonstrated that SLNs had 150% higher C_max_ (~1066 ng/mL) and a 2.8-fold enhanced AUC (5736 ng h/mL), compared with the drug solution (C_max_; 655 ng/mL and AUC; 2067 ng h/mL). The result obtained concluded that SLNs serve as potential drug delivery carriers for enriching drug concentration in topical ocular delivery and could be efficacious in treating endophthalmitis [111].

Despite the application of statins in cardiovascular disease, it is largely explored in the management of age-related macular degeneration. To improve poor aqueous absorption in the ocular region, Yadav et al., investigated atorvastatin (ATS)-loaded SLNs as eye drops for self-use. They developed ATS-SLNs by hot, high-pressure homogenization and characterized in vitro in the ocular application. Their findings revealed that ATS-SLNs were 12-times more bioavailable in the ocular tissues than the conventional eye drop. The stability of the formulation was established as 13.62-times higher, including photostability. The fluorescein-labeled SLNs (F-SLNs) confirmed effective uptake of F-SLNs and prolonged ocular residence upto 7 h [52].

## 6. Inorganic NPs

### 6.1. Mesoporous Silica NP

The silica particle is a highly porous structure that is widely used as a drug delivery carrier in various routes; nevertheless, ocular delivery of silica NP is limited, owing to the toxicity of degraded product of silica as silicic acid, which causes an inflammatory reaction when implanted in the conjunctiva, although silicic acid is naturally present in the human body. To investigate the toxicity, safety, and biocompatibility of silica particles, Paiva et al., developed tacrolimus (TAC)-loaded mesoporous silica NPs functionalized with aminopropyltriethoxysilane (MSNAPTES). They evaluated the cell viability of MSNAPTES and TAC-loaded MSNAPTES (MSNAPTES-TAC) in the ARPE-19, and further, a chorioallantoic membrane (CAM) assay model was employed to show the biocompatibility and safety after intravitreal injection in vivo. Moreover, it was clinically examined based on measurement of intraocular pressure, ERG, and histopathological studies in rats’ eyes [53]. 

The in vitro characterization investigated that the NPs were tagged with functional moiety. The drug-free MSNAPTES and MSNAPTES TAC NPs reported a lack of in vitrocytotoxicity. After the application of NP, no sign ofretinal abnormalities, vitreous hemorrhage in the eye, neovascularization, and retinal detachment were observed during in vivo study. However, follow-up studies on ERGs indicated no changes in the retina cell function after 15 days of intravitreal injection, which were also proved by histopathologic examination, further supporting the biocompatibility, safety, and efficacy of the developed nanocarrier system in ocular therapy. Thus, the findings affirmed the considerable potential of MSNAPTES TAC to transport therapeutics in the ocular delivery for the treatment of eye diseases [53].

Furthermore, another author pointed to safety concerns of intravitreal injection of mesoporous silica NPs in guinea pigs’ and rabbits’ eyes. They establish in vitro cytotoxicity using silicic acid from sol–gel particles on EA.hy926 cells and found dose-dependent cytotoxic effects. They prepared various sizes of silica NPs, out of which 15 μm silica particles with 10 nm pore size were found to be safe in animals’ eyes and were retained there for >2 months. The other preparation of larger pores established a localized, depot formation of sol–gel particles with a variable glassy overcast around them in the aqueous humor, with few inflammatory cells. The inflammatory responses trends were higher with greater pore size particles. The developed sol–gel mesoporous silica particles acquired consistent particle sizes and distinct pores, beneficial for implantation through a fine needle. The optimum formulations could be exploited as nanocarriers in intraocular therapeutic systems, ensuring appropriate drug loading and encapsulation [112].

### 6.2. Gold NPs

Gold NPs typically comprise an inert core of gold and an outer active layer. The particle size distribution is narrow, varying from 1nm to 150 nm. The outer layer of gold NPs could be tuned for the desired ligation with proteins and peptide molecules for site-specific receptor-based targeting of therapeutics, whereas the inner layer of gold particles can safely encapsulate drug molecules [113]. It has been shown that i.v. instillation of gold NPs of appropriate size can cross the blood–retinal barriers and be uniformly distributed end-to-end retinal layers without toxic effect to the cells.

In view of this, Kim et al., demonstrated that i.v. administration of 20 nm size of gold NPs into C57Bl/6 mice enabled passing the blood–retinal barrier, which was well detected around the retinal layers. It was importantly noted that NPs accumulation at the site of retinal cells did not cause any defect in the cell or cell death, compared with the cells lacking NPs. The results indicated that small-sized gold NPs (20 nm) could possibly be employed in the ocular drug delivery and can efficiently penetrate blood–retinal barriers in the ocular disease [54]. Gold NPs as theranostic applications are well discussed in the literature, especially in different cancers in the human body. Mitra et al., designed gold NPs (AuNPs) covered with polyethyleneimine (PEI) and thereby ligated with epithelial cell adhesion molecule (EpCAM) antibody and siRNA molecules. They stated that the gene delivery system was prominently internalized by retinal cells, probably due to the overexpressed receptor cells ensuring significant cytotoxicity to cancer cells. Regardless of significant efforts made on the intraocular cancer therapy, the study relied on in vitro characterization constraints, as well as on mature intraocular cancer animal models [55]. Qiong et al., incorporated bimatoprost-loaded gold NPs, and their uptakeinto contact lens from a soaking solution was investigated; the authors showed improved release kinetics both in vitro and in vivo due to high uptake from soaking solution without causing any alteration in the contact lens, indicating therapeutic efficacy in ocular diseases [114].

## 7. Contact Lenses

To increase the drug contact time in the ocular tissue, ocular lenses are designed with polymeric materials that encapsulate the drug molecules to prolong the therapeutic effect in the local ocular tissues. Contact lenses are thin, curved-like polymeric disks, which are designed in such a way that can comfortably fit into the cornea. Upon insertion into the cornea, the contact lens clings to the cornea due to physical adherence or surface tension. Contact-lens-incorporating drugs have been explicated in the ocular delivery of antihypertensive drugs, antimicrobials, and antihistamines. The contact lens improved the drug residence time in the ocular tissue and tear film, which successively increased drug absorption in the cornea and minimized drug loss via nasolacrimal duct. Usually, the drug is loaded into contact lenses by soaking them in drug solutions. These soaked contact lenses demonstrated higher efficiency in delivering drugs, compared with conventional eye drops [115]. 

To overcome the challenges of drug loss from precorneal surface especially in the treatment of corneal conjunctivitis and keratitis, moxifloxacin (MF) with Dex was designed in a drug-eluting polymeric contact lens for effective drug delivery. The contact lens comprised polymer chitosan, polyethylene glycol, and glycerol. The polymer contact lens was prepared in a combination drug as well as separately. The dual drug-loaded contact lenses were characterized in vitro for surface characteristics, swelling index, thickness, and mucoadhesive property. Furthermore, in vitro release was conducted to investigate release profile behavior, corneal permeation ex vivo, and antimicrobial activity both in vivo and in vitro. The contact-lens-loaded therapeutics was compared with pure drug solutions and commercially available products. The results indicate that drug-loaded contact lenses exhibited higher corneal drug distribution 24 h post incubation, compared with pure drug solutions. Further, in vitro and in vivo investigations showed drug-loaded contact lenses were superior to pure drug solutions. The drug biodistribution indicated adequate drug concentrations in the cornea, aqueous humor, and the sclera using contact lenses, compared with drug solutions. These findings showed that the drug-eluting polymeric contact lens was effective in delivering both the drug MF and DM in the precorneal area and could achieve therapeutic concentrations, suggesting their preferred applications in post ocular operations and eye infections [56].

Kim and Chauhan observed that contact lenses made up of poly (hydroxyethyl methacrylate) exhibited higher Dex absorption, compared with eye drops. However, the developed contact lens had limitations such as poor drug loading and burst release. To further improve the drug release characteristics, particle-laden contact lenses with molecular imprints have been explicated. Particle-laden contact lenses are prepared by dispersing the drug entrapped vesicles—namely, liposomes, NPs, or ME, as well as the material base of the contact lens [57].

In a subsequent attempt, Gulsen et al., designed particle-laden contact lenses for lidocaine delivery in the ocular tissue. They developed lidocaine-loaded ME drops and liposome in a different study and dispersed them in particle-laden contact lenses, which comprised poly-2-hydroxyethyl methacrylate (p-HEMA) gels. Their findings conveyed that lidocaine showed sustained release for upto a period of 8 days. Thus, particle-laden contact lenses are expected to deliver therapeutics for an extended period in the ocular region [58].

## 8. Implants

Ocular implants are fabricated to provide localized controlled release of therapeutics for a prolonged period. The problem arises due to frequent intraocular injection, and related complications can be generally replaced with using intraocular implants. This device is placed intravitreally with a minor incision at pars plana situated between lens and retina. The intraocular implants receive overwhelming interest due to sustained and localized drug release in the ocular disease tissues to attain therapeutic levels, reduce unwanted effects, and also overcome the blood–retina barrier [59]. Many devices to implant in the ocular cavity have been designed and formulated as ocular therapeutic systems for vitreoretinal chronic therapy.

Sheshala et al. developed an investigational implant of biodegradable poly-lactic-co-glycolic acid (PLGA)-injectable, phase inversion, in situ forming system for controlled drug delivery of TA. The implant developed with variable concentrations of TA that ranged from 0.5% to 2.5% *w/w* was dissolved in a solvent and subsequently incorporated into 30% *w/w* of polymer, PLGA (50/50 and 75/25); the resulting solution formed a homogenous injectable preparation. The formulation was evaluated in vitro for the measurement of various parameters. The formulation showed a viscosity of 0.19–3.06 Pa.s, good syringeability, and shear thinning behavior. The microscopic examination indicated that the thickness of the implant depended on time and rate of implant formation, further demonstrating the fast phase inversion. The implant showed 42 days of sustained drug release in the ocular environment. These findings concluded that the PLGA/solvent-based, phase inversion, in situ forming implants can ameliorate the therapeutic treatment outcome in the ocular disease by sustaining the drug release for an extended period of time, thus reducing the dosing frequency of injections [61].

Both biodegradable and nonbiodegradable ocular implants are available as drug-releasing devices. Biodegradable implants are developed using FDA-approved polymers, e.g., polylactic acid (PLA), polycaprolactones (PCLs), PLGA, and polyglycolic acid (PGA) [116]. The polymers used in designing the nonbiodegradable implants are silicone composite, polyvinyl alcohol (PVA), and ethylene-vinyl acetate (EVA). Compared with bio-degradable implants, nonbiodegradable implants lengthen drug release via zero-order release kinetics. 

### Nonbiodegradable Implants

Vitrasert^®^ (Bausch and Lomb Inc., Rochester, NY, USA) is FDA-approved ganciclovir containing Vitrasert^®^ for intraocular implants, with controlled release characteristics used in cytomegalovirus retinitis therapy. The implant is composed of 4.5 mg tablet of ganciclovir encircled by PVA/EVA, which releases drugs for an extended period of 7 months, is available at low cost, and without systemic toxicity. 

Retisert^®^ (Bausch and Lomb Inc., Rochester, NY, USA) is an FDA-approved non-biodegradable implant applied for chronic uveitis therapy and also affects the posterior segment of the eye, bearing silicone-laminated PVA. Fluocinolone acetonide was released from Retisert^®^ in a sustained manner for up to 3 years. The implant had a better effect in reducing the inflammation, preventing recurrences of uveitis, and improvingvisual acuity as well [59,60].

In contrast, another class of implants is largely explored due to the implants’ biodegradable and biocompatible features, apart from showing sustained drug release characteristics. The biodegradable nature of the implant required no surgery for removal, which preferably degrades in the biological milieu, compared with nonbiodegradable implants. PGAs, PLAs, PLGAs, and polycaprolactones are commonly employed polymers for the fabrication of such implants. 

For instance, Surodex™ and Ozurdex^®^ are biodegradable implants designed for intraocular Dex-sustained delivery in the ocular inflammation and macular edema (ME) of the eye. Surodex™ (Allergan, Inc., Irvine, CA, USA) has active ingredients including Dex in PLGA and hydroxypropyl methylcellulose. In postcataract-surgery-linked inflammation, the implant is inserted in the anterior chamber of the eye to control the release of medicaments for a week and to curb inflammation and related problems [59].

Ozurdex^®^ (Allergan Inc., Irvine, CA, USA) was approved by the FDA in June 2009 as another biodegradable intravitreal implant for the treatment of ME. It is based on Allergan’s NOVADUR^®^ technology used in the ocular delivery of Dex. The NOVADUR^®^ system comprises a polymeric PLGA matrix that tardily degrades into lactic acid and glycolic acid and accommodates Dex release for upto 6 months. The clinical trials investigated their efficacy in reversing vision loss in patients and observed improvedsharp vision in eyes associated with ME, i.e., vein occlusion in the retina [59].

The role of small molecules in ocular therapy is apprehended due to the advantages of specificity, transportability, scalability, and efficacy. Ocular drug implants (ODIs) wererecently demonstrated by Sun et al., for the sustained effect of medicine in the ocular cavity. The methodology was based on intravitreal injection of the implant into the small eyes of genetically modified mice. They used biodegradable PLGA as polymer and small molecule cyanine 5.5 as an active entity, in the weight ratio of 95 to 5, and formed Cy5.5-PLGA that showed sustained drug release. Further, the authors investigated the efficacy and ocular bioavailability of ODIs and thereby developed a robust, economic, and minimally invasive technique known as “mouse implant intravitreal injection” (MI3) into the mouse eyes. This procedure could be clinically translated into the treatment of human eye diseases [117].

## 9. Recent Advancements in Ocular Delivery

### 9.1. Nanofiber Inserts

The large surface-area-to-volume ratio is an important feature that draws attention to nanofiber-based ocular drug delivery. The nanocarrier system can be effectively administered into the conjunctival sac and contacts well with the ocular segment. Nanofibers could overcome the limitation of eye drops with respect to cul-de-sac volume (∼30 μL). Many researchers designed multilayered nanofibers inserted with an intermittent layer of therapeutics using various polymers including Eudragit RL 100 [118].

Mirzaeeiet al., prepared a nanofiber insert with a modified electrospinning technique, to improve ocular residence time and prolong drug delivery of ofloxacin (OFX) from the nanofiber insert. The chitosan/polyvinyl alcohol (CS/PVA) nanofiber layered in Eudragit RL100 was fabricated by crosslinking technique intended for conjunctivitis. Firstly, the electron microscopy showed that the average fiber diameter of 123 ± 23 nm for a single electrospun nanofiber lacked crosslinking. Secondly, a crosslinked single electrospun nanofiber hadan average diameter of 159 ± 30 nm. Antimicrobial efficacy in bothsingle-spun and multispun nanofibers demonstrated a potential zone of inhibition against *S. aureus* and *E. coli*. The ofloxacin release from nanofiber inserts on the rabbit eye affirmed a sustained release behavior for up to 96 h. It is further found that the crosslinked nanofibers could reduce the burst release of drugs in single- and multi-layered nanofibers. The animal study exhibited that the nanofibers showed a 9–20-fold increase in bioavailability, compared with drug solutions. Thus, the demonstrated potentiality of the electrospun nanofiber approach to prolong the drug release in ocular segments was evidently promising [62]. 

Electrospinning is a diversified technique mainly formed by electrostatic repulsion charge surface, which draws nanofiber from a viscoelastic liquid.It is composed of polymers, ceramic nanomaterials, and nanosized molecules in this combination. This technique successfully developed nanofibers in vitro. The nanofiber produced by the electrospinning technique is characterized by a solid smooth surface with a number of branches as secondary structures. It has a hollow- or core-sheath-like structure with numerous pores. The existing large surface area on both the outer surface and anterior part can be modified by a number of molecular species, proteins, ligands, or NPs during the processing of such technique [63].

Researchers’ interest in the use of electrospun nanofibers in ocular therapeutic platforms is tremendously increasing, as the system may accommodate the eye surface and provide controlled drug release. Exploring this technique, Gimaudo et al., designed a novel ocular insert consisting of hyaluronan (HA) nanofibers in a combined delivery of ferulic acid (FA), an antioxidant, and an antimicrobial peptide (ε-polylysine, ε-PL). The designed fibers were obtained with diameters of ~100 nm, containing PVP 5%, HA 0.8% *w*/*v*, PVP 10%, and HA 0.5% *w*/*v* in an organic and aqueous mixture. The increase in PVP concentration led to an increased thickness of ~1 µm. Nanofibers crosslinking with ε-PL, blank, and FA-loaded inserts demonstrated average thicknesses of 270 ± 21 µm and 273 ± 41 µm, respectively. The insert enabled the complete release of ε-PL, both from blank and drug-encapsulated inserts, within half an hour under dissolution medium. The FA-loaded inserts showed considerable antimicrobial efficacy against *P. aeruginosa* and *S. aureus* [119].

Khalil et al. developed an alternative to conventional eye drop for the first time. They designed a single-dose mucoadhesive biodegradable polymeric-multilayered NPs-in-nanofibers (NPs-in-NFs) matrix as an ocular insert of azithromycin. The ocularinsert was prepared with electrospinning technique using polyvinylpyrrolidone and investigated their efficacy in vitro against bacterial infection in the eye. The drug release and permeation profile established that ocular insert could render controlled drug release upto 10 days. The authors concluded that the incorporation of NPs into NFs could achieve many advantages such as increased ocular residence due to better contact with conjunctiva, accurate dosage, prolonged drug release, reduced dosing frequency, reduced systemic side effects, and improved bioavailability [64].

Taghe et al., developed azithromycin Eudragit^®^ L100 NPs for sustained drug release and improved ocular performance. The NPs were prepared by solvent diffusion method with sonication technique. The drug-loaded Eudragit^®^ L100 NPs were plasticized using polyvinyl alcohol (PVA) solutions and investigated in vitro. Moreover, azithromycin ocular insert (film) was developed using a solvent casting method by applying a solution of cellulose derivates hydroxypropyl methylcellulose (HPMC) or hydroxyethyl cellulose (HEC). The final preparation of NPs had entrapment of 62.167 ± 0.07%, a particle size of 78.06 ± 2.3 nm, a surface charge of −2.45 ± 0.69 mV, and a polydispersity index of 0.179 ± 0.007. The developed inserts expressed antimicrobial effects on *S. aureus* and *E. coli* cultures. The release profile of the drug was initially immediate, followed by sustained release, and thus significantly prolonged the release of drug from inserts, compared with the drug solution. This trans-corneal ex vivo study demonstrated higher corneal drug permeation, compared with the drug solution. Therefore, the ocular insert offered a promising method for the delivery of azithromycin in the ocular chamber as treatment of eye inflammation and infections [65].

### 9.2. Microneedles (MNs)

MNs offer a minimally invasive drug delivery approach in localized targeting to the posterior ocular tissue, with high precision and accuracy, compared with hypodermic needles. The MN-based drug administration may repress complications that arise in intravitreal injections, which may lead to retinal breakups, hemorrhage, cataracts, eye inflammation, and infection. This technique has the potential to improve vision jeopardized by posterior ocular diseases linked to age-related macular degeneration, posterior uveitis, and diabetic retinopathy [66]. The strategic drug delivery approach using MNs may overcome the blood–retinal barrier and prominently deliver therapeutics inside the retina or choroid. 

MNs have traditional designs and are capable of penetrating the limited depth of the sclera, thereby preventing major damage to the deep ocular tissues. Using this needle, it is easy to deposit therapeutics or drug delivery systems into the sclera or in between sclera and choroid spaces, the so-called suprachoroidal space. Micropuncturing the sclera layer may facilitate higher deposition of drug molecules or drug carriers in the sclera, and more drug diffusion into the deeper ocular tissues may result [120]. Jiang et al. investigated the surface-coated MNs with drugs in cadaver eyes to study the sclera surface penetration by MNs, sulforhodamine dissolution, and release behavior from MNs into the intrascleral region. The results showed that the surface layered drug instantaneously dissolves in the sclera tissue, intimating high sulforhodamine deposition in the sclera via MNs hole [67].

In another attempt, Jiang et al., prepared and evaluated MNs’ performance as complements to drug solution infusion from nanocarriers into the sclera tissues. Using MNs, the authors enabled the infusion of ~10–35 μL fluid directly into the sclera tissues. They further investigated MNs for delivery of nanosuspensions and microparticles in the sclera layer. The outcome of their study revealed that the vacuous MNs loaded with micro/NPs are better routes for drug delivery into the sclera with minimal invasiveness [68]. 

In 2016, Thakur et al., studied the dissolving nature of MNs for increasing the bioavailability of the macromolecules in the ocular tissue. The fabricated MNs comprised polymers and PVPs of different molecular weights (MWs), with three molecules of high MW fluorescein sodium and fluorescein isothiocyanate–dextrans (MW ranges between 70 k and 150 k Da). The dimension of MNs was a height of 800 μm and a base diameter of 300 μm, with model drugs, which were prepared and characterized in vitro in terms of braking forces, insertion forces (in the sclera and the cornea region), penetration depth using OCT and confocal imaging, drug release time, and permeation studies. The results showed that the high-MW PVP-fabricated MNs could withstand greater forces with a petty reduction in needle height. Polymer MNs showed fast dissolution within 180 s depending upon PVP’s MW. In vitro studies demonstrated high permeation of macromolecule across the scleral and corneal tissues, compared with aqueous solutions. Interestingly, macromolecules formed depots inside the ocular tissue, as indicated by confocal images, and helped in sustained permeation. Biomaterials used in the fabrication of MNs were biocompatible and had the potential for drug retention in specific ocular tissues. The collective outcomes showed that the designed MNs are minimally invasive, rapidly dissolving devices that have high insertion forces to deliver macromolecules through the transscleral route in eye diseases [121].

Considering the critical challenges imposed by the ocular barrier in overcoming ocular diseases and injuries in eyes resulting in significant clinical impairment, Thanet al. strategically developed an eye patch furnished with a layout of detachable microneedles. The developed microneedles could enable deeper penetration into the ocular surface tissue and had a controlled drug release from these microimplanted reservoirs. The biphasic drug release pattern from multilayer microreservoirs enhanced therapeutic efficacy. The corneal neovascularization model associated with eye disease indicated antiangiogenic characteristics of the monoclonal antibody (DC101) microneedle, which can reduce ~90% of the neovascular region. Moreover, the onset of diclofenac, an anti-inflammatory agent followed by a sustained release of DC101, provided synergistic therapeutic efficacy. The application of microneedle as an eye patch is minimally or noninvasive, easy to administer, and ensures patient compliance. Thus, the intraocular therapeutic delivery approach could be promisingly efficacious against several eye diseases [69]. A brief summary of recent patents on ocular disease therapy is included in Table 2.

## 10. Recent Patents on Ocular Disease Therapy

US Patent No. 11,000,509 disclosed the composition and methods of formulation of phentolamine in kits for ameliorating visual operation. The routine instillation of ophthalmic phentolamine solution improvedvisual acuity and minimized the appearance of redness in the eye. The composition of invention benefitted the patients enduring miserable visual performance both during the day and night time [122]. Another patent by the same inventor disclosed the composition and method for the preparation and kits of phentolamine, an alpha-adrenergic antagonist intended for monotherapy or used as a combination therapy in the treatment of presbyopia, mydriasis, and/or other ocular complications [123]. A similar patent on an aqueous ophthalmic solution of phentolamine was mentioned in application number 11090261, credited to Ocuphire Pharma, Inc. The inventor of the patent, Alan Meyer, disclosed the ophthalmic solutions of phentolaminemesylate in combination with pharmaceutically acceptable additives such asmannitol, sodium acetate, and water as a medical kit for improving visual performance [138].

Babizhayevet al. invented a patent to treat eye diseases with an aqueous ophthalmic preparation of *N*-acetylcarnosine, a *N*-acetylcarnosine derivative or a pharmacologically acceptable salt of *N*-acetylcarnosine, with cellulose imparting the intraocular absorption of the said compound. The therapeutics were incorporated into a polymeric hydrogel contact lens, and ocular insert for topical instillation, for effective therapy of corneal problems associated with glaucoma and blurred vision [124]. Application KR20200000395A disclosed the composition of a nanoemulsion comprising an active ingredient cyclosporine in a highly dissolve state and an emulsifier in an aqueous vehicle. The nanoemulsion established desired particle size of 100 nm and narrow particle distribution. The preparation showed efficacy against ocular problems such asdry eye syndrome, conjunctivitis, and tingling sensation [125].

Moreover, a patent disclosed a composition of ophthalmic nanoemulsion having cyclosporine (0.01–0.1%), propylene glycol monocarprylate (0.1–2.5%), poloxamer (0.5–15%) glycerine, (0.1–10%), and chitosan or carbomer (0.1–5%). The preparation technique involves drug dissolution in propylene glycol monocarprylate leveled as a solution I, followed by dissolving the poloxamer, glycerin, and chitosan or carbomer in purified water to obtain solution II, and ultimately, mixed both solutions I and II whileadjusting pH by 7.2–7.6. This invention was used for dry eye syndrome [126]. 

A patent disclosed a composition of eye drops of a nanoemulsion and their manufacturing aspects. The composition consists of cyclosporine, a solubilizing agent, a stabilizer, and a solvent. The droplet size of the nanoemulsion was established at 20 nm or less. The nanoemulsion eye drop improves stability and patient compliance, used in dry eye syndrome [127]. Novel active compound EP4 agonists havebeen successfully invented for lowering intraocular pressure, glaucoma, and ocular hypertension, and their efficacy clearly proved in animal models is mentioned in [128]. 

Further, a nanomicelles preparation credited to Mitra and Weiss consisting of polyoxyl lipid (fatty acid) and/or a polyalkoxylated alcohol has been disclosed in the patent US8980839B2 [129]. Similarly, a liposomal preparation of eye drops was invented by Kato et al. for the treatment of dry eye syndrome [130]. An invention provided herein is an ophthalmic formulation having a polyoxyl lipid or fatty acid, and/or a polyalkoxylated alcohol in nanomicelles. It also includes the method of treating or preventing ocular diseases or conditions. The preparation is used in glaucoma and retinal diseases [131].

The use of gold cluster or gold-cluster-containing substance in the preparation of drugs for preventing and/or treating glaucoma has been disclosed in WO2018095429A1 [132]. A chitosan- or hyaluronic-acid-based nanocrystalline eye drop and its application in congenital macular degeneration has been revealed in a patent CN110664757A. The developed formulation may work based on the designed targeting approach to endothelial growth factor or a platelet growth factor receptor. The medicament could manage the penetration of the ocular barriers through passive targeting or intercellular pinocytosis approach, thereby providing effective drug concentration in the cul-de-sac cavity [133]. 

US patent US9295693B2 disclosed a composition of water-soluble graft copolymer poly(l-Lysine)-graft-poly(ethylene glycol) coated with sterically stabilized PEG coatings, which is good for binding with biological surfaces. The composition is used in dry eye syndrome and contact lens intolerance therapy [134]. A pharmaceutical composition of cell-penetrating peptides has potential permeation ability in the ocular tissues without observing any toxicity. The peptide is a good absorption enhancer and is givennoninvasively in the ocular chamber, therefore improving the ocular bioavailability. Furthermore, the invention is suggested as an alternative to ocular injection and improved patient compliance in intraocular diseases treatment [135].

A liposomal preparation of corticosteroid is given locally in the inflammatory lesion in the ocular tissue. Further, the composition of liposome with uncharged lipid or Pegylated liposome is used for the treatment of inflammation lesion with the application of once in two weeks [136]. The topical formulations of olopatadine for allergic or inflammatory disorders of the eye and nose are disclosed in another patent. The composition of formulation has olopatadine (0.17–0.62% *w*/*v*) with a sufficient quantity of polyvinylpyrrolidone or polystyrene sulfonic acid as a stabilizer [137].

## 11. Conclusions and Future Prospects

Drug delivery challenges due to the various ocular barriers confoundpotential setbacks for scientists working on targeted drug delivery in the ocular tissues. Nevertheless, considerable efforts have been made so far, and the trends still continue in seeking ocular drug delivery, seeking efficacious, safe, and biocompatible therapeutic drug delivery approaches. At present, researchers working in this domain strive to enhance the performance of the conventional formulation in vivo. Many drug delivery carrier systems with nanotechnology applications are being designed and formulated at a large scale, including polymer NPs, solid lipid NP, lipidic vesicles, micelles, micro- and nanoemulsions, nanosuspensions, microneedles, and dendrimers. For instance, several ocular drug delivery studies are only limited to in vitro performances, and explorations of in vivo studies using ocular models of cell lines may further facilitate the development of precise data at the preclinical and clinical stages.

Nanotechnology-based drug delivery systems so far have been useful in reducing toxicities, multiple dosing, dose-related undesired effects, and fluctuations in drug plasma concentration, associated with a traditional drug delivery system. However, research on ocular therapeutic systems still suffers from a large gap, and the design process of a novel carrier that could be nontoxic, biodegradable, biocompatible, and efficacious in mitigating both the posterior and anterior segments of the eye disease is underway. The current attempts and prospects made by scientists regarding ocular therapeutic systems may translate nanomedicine into clinics with promising precorneal residence time and high drug accumulation in targeted ocular tissues, reduced frequency of administration, and good bioavailability. Finally, combining both the therapeutic and diagnostic agents in one nanocarrier system may bring better visual acuity in ocular therapy.

## Figures and Tables

**Table 1:** Nanotechnology-based novel drug carrier system in the treatment of ocular disorders.

Carrier System	Payload	Composition	In Vitro Characteristics	In Vivo Observations	References
Microemulsion (ME)	TA	The ME comprised a ratio of oil (Capmul MCM C8): surfactant (AccononMC8-2): cosurfactant (Transcutol): water of 5:35.5:4.5:55. Thereafter, ME was PEGylated using 1, 2-distearoylphosphatylethanolamine-polyethyleneglycol 2000 (DSPE-PEG 2000).	The ME was small in size, uniform, and transparent. The average size obtained was 157.720 ± 17.85 nm.	The prepared PEGylated ME observed stable, homogenous, and nonirritant to the eye after animal study and had the potential to target the posterior segment of the eye after topical administration.	[2]
Polymer micelles	Netilmicin and Dex	Nanomicelles using copolymers of polyhydroxyethylaspartamide (PHEA) and pegylated PHEA for topical anterior segment drug delivery.	In vitro permeation study on conjunctival and corneal epithelial cells performed using drug-loaded polymer micelle (PHEA-C_16_ and PHEA-PEG-C_16_).	The drug-loaded polymeric micelles increased 40% ocular bioavailability, compared with Dex suspension.	[33]
NPs	Melatonin	PLGA-PEG NPs	Decreased surface charge of in PLGA-PEG than the PLGA alone accorded good and enhanced interaction of NPs with eye surface.	The effective lowering of intraocular pressure (IOP), compared with melatonin PLGA NPs and drug solution with the same concentrations, was observed in the rabbit’s eye.	[34]
NPs	Dex	DEX-loaded poly(lactic acid–co-glycolic acid) NPs (DEX-NPs)	DEX-NPs showed narrow size distribution with a particle size diameter of 232 ± 5.4 nm and a polydispersity index of 0.19. The drug encapsulation efficiency was 56.0%. The constant drug release of 97% was observed for upto 35 days.	Ophthalmic investigations based on fundus examination, IOP measurement, and ultrasonography have shown no abnormalities till 50 days after DEX-NPs instillation in the rabbit’s eye. The intravitreal injection provided sustained release of drugs in the posterior segment of the eye disease. The DEX-NPs showed sustained drug release for 50 days in the vitreous humor, and a mean concentration of 3.85 mg/L^−1^ was constantly found for >30 days.	[35]
Hybrid NPs and nanosheets	Pirenoxine sodium(PRNs)	A multifunctional organic–inorganic hybrid NPs and nanosheets, new chitosan–glutathione–valine–valine-layered double hydroxide (CG-VV-LDH) nanosheets.	LDH nanosheets showed a mean size of 47.5 ± 12.1 nm and polydispersity index (0.210 ± 0.021), and zeta potential ~ (35.4 ± 0.9) mV. The burst release of PRN solution was ~100% in 2 h, while 74.6% of PRN release was reached in a sustained manner from CG-VV-PRN-LDH nanosheets over a 12 h time period.	The epithelial cell (HCEpiC) and retinal pigment cell (ARPE-19) uptake demonstrated complete cell internalization via clathrin and endocytosis pathways. The active transport of PepT-1 is implied in the CG-VV-LDH NPs and CG-VV-LDH cell internalization process. The CG-VV-PRN-LDH NPs eye drops werepermeated ~5.2fold higher than the marketed product. The CG-VV-LDH NPproveda promising nanocarrier in ocular disease therapy, while CG-VV-LDH nanosheets extended the precorneal retention and were found suitable for ocular surface diseases.	[36]
Nanosuspensions	Dex, prednisolone, and hydrocortisone	Nanosuspensions, suspension, and solutions state	-	The crystalline drug suspensions of these drugs (Dex, prednisolone, and hydrocortisone) enhanced the rate and extent of absorption, as well as the intensity of drug action in ophthalmic delivery. Despite these, all preparations were enable to lower the high IOP, compared with drug solutions.	[37]
Nanosuspensions	Flurbiprofen	Eudragit RL 100	The developed nanosuspension had spherical particle shape, particle diameter around 100 to 200 nm, surface charge ranges from +6.6 ± 2.2 to +19.0 ± 3.1 mV, and drug encapsulation recorded in between 54.67 ± 3.4 to 90.32 ± 3.2%.	The nanosuspension underwentsustained release of drug (60%) over 12 h, compared with marketed preparation (Flur eye drops). The in vivo study in animals reported it to be nonirritant and safe based on histopathological studies.	[38]
Liposomes	Besifloxacin	Liposomes developed with phosphatidylcholine (LP PC) or phosphatidylcholine and spermine (LP PC: SPM)	The mean vesicle diameter of liposome (LP PC) was found to be177.2 ± 2.7 nm, with a surface charge of −5.7 ± 0.3 mV, and for liposome (LP PC: SPM), the mean diameter and zeta potential were observed as 175.4 ± 1.9 nm and +19.5 ± 1.0 mV.	The MIC and MBC of the liposomal formulation werelower than the marketed preparation, Besivance against *P. aeruginosa*.	[39]
Liposomes	Latanoprost	DPPC (di-palmitoyl-phosphatidyl-choline), organic solvent chloroform:methanol mixture (2:1)	The average size and least PDI of the vesicle were reported around 100 nm and 0.11. The vesicle size may vary depending upon the drug/lipid ratio.	No increase in inflammation or vascularity was noted after subconjunctival injection. The single subconjunctival injection of liposomes demonstrated lowering IOP due to sustained ocular drug delivery and was a suitable alternate option to the conventional eye drops.	[40]
Liposome complex	DNA	Liposome–protamine–DNA complex (LPD), a biomimetic virus modified with cell-penetrating peptides	-	The outcomes of in vivo study demonstrated that modified liposome expressed effective Rpe65 gene delivery in a specific and further alleviated long-term expression of the Rpe65 gene in the RPE disease model resulting in rectification in blindness.	[41]
Cationic and anionic liposomes	Acyclovir	Liposomes prepared using stearylamine (cationic) and dicetylphosphate as anionic charge-inducing agents	-	The observed concentration of the drug in the cornea for drug solution, positive (+) and (−) charged liposomes were 253.3 ± 72.0, 1093.3 ± 279.7, and 571.7 ± 105.3 ng/g. The absorbed drug concentration from positively charged liposomes was 2-times higher than (−) charged liposomes and 5-times higher than the drug solution.	[42]
Dendrimer	Pilocarpine nitrate and tropicamide	PAMAM solutions, fluorescein dye and carbopol (0.2% *w*/*v*)	-	The PAMAM dendrimer with carboxylic and hydroxyl surface groups showed longer ocular residence time than dendrimer solutions. The residence time of PAMAM dendrimer in the cornea was size- and molecular-weight dependent.	[43]
Dendrimers	brimonidine tartrate	Polyamidoamine dendrimers	The drug release kinetics and safety concerns of nanofiber mats were securely investigated in vitro.	The dendrimer did not show toxicity or irritation at the therapeutic dose in the animal. The single-dose administration of DNF indicated significant improvement in efficacy, compared witha drug solution, for 3weeksprovedthat dendrimer nanofibers can effectively be given in glaucoma therapy.	[44]
Nanoliposomes dispersed within thermo-sensitive hydrogels	Senicapoc	DPPC (Carboxyfluorescein) liposomes, Pluronic F-127 polymers, organic solvents (chloroform and methanol)	In vitrostudy has shown that Senicapoc was sustainably released from DPPC liposomes for a prolonged time of 28 days and achieved a cumulative release of 81.2 ± 1.7%.	In vivo results showed that Pluronic F-127 hydrogel at (24 *wt %*) concentration enhanced nanoliposomes residence time on the surface of the eye and thus increased bioavailability. Further, senicapoc sub-conjunctival injection maintained the drug concentration upto 24 h.	[45]
TAC-SLNs ISG	Tacrolimus	Compritol^®^ 888 ATO (0.25% *w*/*v*) and GMS (2% *w*/*v*), TAC SLNs and Poloxamer 188 (12% *w*/*v*)/Poloxamer 407 (26% *w*/*v*).	The probe sonicated particle of TAC-SLNs ISG had particle sizes of 122.3 ± 4.3 nm. TAC-SLNs-ISG showed a pseudoplastic flow.	In vivo study showed that eye drops and TAC-SLNs had C_max_ 4657.7 ng/mL and 1892.6 ng/mL within 30 min, while TAC-SLNs-ISG had achieved 2132.3 ng/mL within 2 h. The AUC_0–t_ of TAC-SLNs-ISG and TAC eye drops were 590,355.9 and 222,382.5 ng.min/mL, i.e., 2.65-folds higher for TAC-SLNs-ISG than for TAC eye drops.	[46]
In situ gel with TL-NPs	Tranilast	Ophthalmic TL-NPs preparation with different percentages of methylcellulose.	The ophthalmic TL-NPs preparation yielded an average particle size of ~93 nm using MC (0.5–3%) or without using MC (0.5–3%).	The gel preparation TL-NPs with 0.5 and 1.5% MC improved the preconjunctival contact time of the drug, resulting in higher drug contents found in the cornea and conjunctiva after topical instillation, compared with TL-NPs with or without 3% MC.	[47]
Nanocapsule	Tacrolimus	Polymer PLGA of MW 100 and 50 kDa; Tween^®^ 80; Cremophor^®^ EL Lipoid^®^; E80 Solutol^®^ HS15 and PVA	Agood lyophilization was observed for PLGA: HPβCD ratio of (1:10). No significant differences were observed in sizes (143.9 and 172.8 nm) incurred before and after this process with a PDI value of 0.2.	The ex vivo corneal permeability established the PLGA NCs retention and permeation in the cornea by >40-fold higher than drug solution, probably owing to smaller particles entrapment in the tight junction of corneal epithelial cells. The PLGA NCs showed a superior anti-inflammatory effect on the anterior chamber LPS-induced keratitis model in comparison with the drug in oil solution.	[48]
Microspheres and nanospheres	Tyrosine kinase inhibitor	PLGA microspheres and nanospheres	In vitro characterization of microspheres and nanospheres revealed particle sizes of ~2.6 μm and ~360 nm.	The intravitreal injection led to the generation of optic nerve within two weeks. Further, nanospheres were found superior tomicrosphere in regrowth of the optic nerve.	[49]
Nanosphere colloidal suspensions	Acyclovir	PLA nanospheres were modified with the PEGylation technique.	-	The high molecular weight polymer led to reduced nanosphere size, and the PEGylated formulation showed sustained drug release and improved pharmacokinetics, well tolerated in the eye. The efficacy of PEGylated PLA nanospheres was significantly higher than the PLA nanosphere.	[50]
Solid lipid NPs	Natamycin	The formulation comprised a solid lipid (4–10% *w/w*), a surface-active agent, Precirol ATO 5^®^ as Pluronic f68 (3–7% *w*/*w*), and a sonication frequency (40 to 80 kHz).	The optimized formula of SLN has been revealed to be 42 r.nm (radius in nanometers), with a surface charge of 26 mV, and EE% reached ~85%. SLN formulation achieved >90% of drug release during 10 h. A corneal permeation study indicated the permeability coefficient (Papp) and steady-state flux (Jss) reached 11.59 × 10^−2^ cm h^−1^ and 3.94 mol h^−1^, compared with 7.28 × 10^−2^ cm h^−1^ and 2.48 mol h^−1^ of the drug solution.	Antifungal activity indicated the increased zone of inhibition was 8 and 6 mm against Aspergillus fumigatus (ATCC 1022) and a clinical isolate of Candida albicans, respectively. The MIC value was reduced to 2.5-times against each strain of fungus.	[51]
Solid lipid NPs	Atorvastatin	The formulation is composed of Comprise^®^ 888 ATO (lipid), ATS, and PEG 400, P188 and P 90H.	The ATS-SLNs havespherical shapes, and the average particle size and PDI are 256.3 ± 10.5 nm and 0.26 ± 0.02.	The finding suggested that ATS-SLNs showed 12-times higher bioavailability than plain drugs in the ocular tissues. The stability of the formulation was found to be 13.62-times higher including photostability, compared withthe drug solution. F-SLNs haveshown effective uptake and prolonged the ocular residence time upto 7 h.	[52]
Silica NPs	Tacrolimus	Silica NPs functionalized with aminopropyltriethoxysilane (MSNAPTES)	The cytotoxicity of developed formulation MSNAPTES and MSNAPTES-TAC in ARPE-19 cells was reported to be dose dependent. The chorioallantoic membrane (CAM) assay model investigated biocompatibility and safety in vivo after intravitreal injection, with a clinical assessment of intraocular pressure and fundus ophthalmoscopy, electroretinography (ERG), and histologic studies in rats’ eyes.	No alteration in retinal cells function was observed after 15 days of intravitreal injection indicated by ERGs and by histopathological studies of rats’ eyes. The 15 μm silica particles with 10 nm pore sizeswerefound to be safe in the animals’ eyes and retained there for >2 months.	[53]
AuNP	-	Gold NPs obtained a size of 20 nm.	-	After injecting AuNP into C57BL/6 mice retinal cells lacking 100 nm particles, on the other hand, 20 nm particles were well detected and distributed through the BRB in the retinal layers. The percentage distributions of these 20 nm particlesin the retina were 75 ± 5% in retinal neurons, 17 ± 6% in endothelial cells, and 8 ± 3% in peri-endothelial glial cells, where the NPs bounded onto the membrane.	[54]
AuNP	siRNA	AuNPs are covered with polyethyleneimine (PEI) and ligated with antibodies, siRNA, and epithelial cell adhesion molecule (EpCAM).	The average size recorded is 28 nm using an AuNP:siRNA weight ratio of 3.	No cytotoxicity to the corneal cells was reported using AuNP-PEI NPs, indicating nanomaterial was safe and suitable for siRNA delivery inocular complications. Moreover, PEI-crowned AuNPs with EpCAM siRNA were internalized prominently in Y79 cells, as shown in fluorescenceand flow cytometry studies, and resulted in significant apoptosis of Y79 cells.	[55]
Contact lens	Moxifloxacin (MF) and Dex	The contact lens comprises polymer chitosan, polyethylene glycol, and glycerol.	Drug-loaded contact lenses exhibited higher corneal drug distribution post 24 h of the incubation period, compared with pure drug solutions.	Both invitro and in vivo investigations showed the drug-loaded contact lens was superior to pure drug solution.	[56]
Contact lens	Dex, Dex 21-disodium phosphate (DXP), and Dex 21-acetate (DXA)	Poly(hydroxyethyl methacrylate) (PHEMA) contact lenses	The transport of DX and DXA is diffusion limited, with diffusivities of 1.08 × 10^−11^ and 1.16 × 10^−11^ m^2^/s, predicted using the transport model.	The contact lenses of these drugs have shown much higher bioavailability than eye drops. Further, among these drugs, the DXA has shown the highest bioavailability.	[57]
Contact lens	Lidocaine	Dimyristoyl phosphatidylcholine (DMPC) liposomes in poly-2-hydroxyethyl methacrylate (p-HEMA) hydrogels	-	Liposome-incorporated p-HEMA gels are transparent and enable drug release for upto ~8 days.	[58]
Biodegradable Ozurdex^®^	Dex	PLGA polymer matrix	PLGA polymer matrix degrades into lactic acid and glycolic acid and accommodates Dex release for upto 6 months.	The clinical investigation revealed their efficacy in reversing vision loss patients and improving sharp vision in eyes associated macular edema, i.e., vein occlusion in the retina.	[59]
Nonbiodegradable Vitrasert^®^	Ganciclovir	Ganciclovir encircled by PVA/EVA.	Vitrasert^®^ released the drug for an extended period of 7 months.	-	[59,60]
Nonbiodegradable Retisert^®^	Fluocinolone acetonide	FDA approved in 2005, a silicone laminated PVA or ethylene-vinyl acetate (EVA).	Fluocinolone acetonide was released from Retisert^®^ in a sustained manner for up to 3 years.	-	[59,60]
Ocular implant	Triamcinolone acetonide (TA)	Implant developed with variable TA concentrations of 0.5%, 1%, and 2.5% *w*/*w* were dissolved in *N*-methyl-2-pyrrolidone (NMP) solvent and thereafter added into 30% *w*/*w* PLGA (50/50 and 75/25) polymer to develop homogenous injectable prepare.	The implant showed good syringeability and rheological features, as well asshear thinning properties. The preparation was easy to administer due to free flowing. The implants established sustained release of drug for more than a month.	The PLGA/solvent-based phase inversion in situ forming implants can ameliorate the therapeutic treatment outcome in the ocular disease by improving drug release for extended periods and reducing the frequency of injections.	[61]
Nanofiber insert	Ofloxacin	Chitosan/polyvinyl alcohol (CS/PVA) nanofiber had layered in Eudragit RL100 fabricated by crosslinking technique intended for conjunctivitis.	The average diameters of single electrospun nanofiber and crosslinked were 123 ± 23 nm and 159 ± 30 nm.	The antimicrobial efficacy of both single-spun and multi-spun nanofiber showed an enhanced zone of inhibition against S. aureus and E. coli. The ofloxacin release from nanofiber inserts on the rabbit eye was detected for upto 96 h. The in vivo study showed 9–20-folds higher bioavailability, compared withthe drug solution.	[62]
Electrospinning nanofiber	Ferulic acid (FA)	Hyaluronan (HA), ferulic acid (FA), an antioxidant and an antimicrobial peptide (ε-polylysine, ε-PL), and polyvinylpyrrolidone (PVP).	Fiber consisted of PVP 5%-HA 0.8% *w*/*v* and PVP 10%-HA 0.5% *w*/*v* obtained with diameters of ~100 nm. The crosslinked nanofiber with ε-PL, blank and FA-loaded inserts demonstrated average thickness of 270 ± 21 µm and 273 ± 41 µm, respectively.	The insert showed complete release of ε-PL both from blank and FA-loaded inserts within 30 min. The FA-loaded inserts were shown improved antimicrobial efficacy against *P. aeruginosa* and *S. aureus*.	[63]
Polymeric nanofiber	Azithromycin	The ocular-insert was prepared with an electrospinning technique using polyvinylpyrrolidone.	The in vitro performances established that the ocular insert could render controlled drug release upto 10 days.	AZT-loaded NPs-in-NFs has shown increased ocular residence, reduced systemic side effects, and improved bioavailability.	[64]
Ocular insert	Azithromycin	Drug-loaded Eudragit^®^ L100 NPs with plasticizer, polyvinyl alcohol solutions. The drug-loaded ocular film was prepared with the solvent casting of cellulose derivates hydroxypropyl methylcellulose (HPMC) or hydroxyethyl cellulose (HEC) solutions.	The preparation of NPs had particle size 78.06 ± 2.3 nm; drug entrapment 62.167 ± 0.07%, surface charge −2.45 ± 0.69 mV, and polydispersity index 0.179 ± 0.007. The drug release from the insert was burst, followed by sustained release, which was significantly higher than the drug solution in the rabbit eye. The trans-corneal drug permeation was reportedly higher than the drug solution.	The developed inserts haveshown antimicrobial effects on *S. aureus and E. coli cultures*.	[65]
MNs	fluorescein sodium and fluorescein isothiocyanate–dextrans	The developed MNs have different molecular weights (MWs) polyvinylpyrrolidone (PVP), fluorescein sodium, and fluorescein isothiocyanate–dextrans (MW in between 70 k and 150 k Da).	Dimensionally MNs with a height of 800 μm and base diameter 300 μm, with model drugs, were prepared and characterized in vitro related to braking forces, insertion forces (in the sclera and cornea region), penetration depth using OCT, and confocal imaging.	The high-MW PVP-fabricated MNs could withstand greater forces. The polymer MNs expressed rapid dissolution within 180 s and varied with PVP’s MW. In vitro demonstrated the high permeation of macromolecule across scleral and corneal tissues relative to aqueous solutions.	[66]
Microneedle (MNs)	sulforhodamine	-	-	High dissolution and release behavior from MNs into the intrascleral region.	[67]
MNs loaded micro/NPs, and fluorescent-tagged NPs	sulforhodamine	-	The microneedle retraction with 200–300 μm rendered 10–35 μL infusion fluid in the tissue.	Better drug delivery in the sclera with minimal invasiveness.	[68]
MNs eye patch	monoclonal antibody (DC101), diclofenac	Hyaluronic acid (HA) and crosslinked methacrylated HA.	The contact eye patch is developed with an array of self-implantable micro-drug-reservoir. The patch hasan arrangement of pyramid-shaped MNs with a tip diameter of ~10 µm, height ~500 µm, base width ~250 µm, and inter-needle spacing of ~400 µm.	The developed MNs could enable a deeper penetration to the ocular surface tissue and had control over the drug release from these micro-implanted reservoirs. The corneal neovascularization model in eye disease indicated MNs can reduce ~90% neovascular region.	[69]

**Table 2:** Recent patents on ocular disease therapeutics.

Patent Application	Title and Year	Description	Clinical Use	References
US Patent No. 11,000,509	Methods and compositions for daily ophthalmic administration of phentolamine to improve visual performance (2021)	The invention providedthe composition, methods, and phentolamine in kits for ameliorating visual operation such as visual acuity by the routine administration of ophthalmic phentolamine solution in the patient eye at bedtime for broad time, which minimizes the appearance of redness in the eye during daytime or waking time. The composition of invention benefitted the patients enduring poor visual performance both during the day and night time.	Visual impairment	[122]
US Patent No. 10,993,932	Methods and compositions for treatment of presbyopia, mydriasis, and other ocular disorders (2021)	The invention relates to the composition and method for the preparation, and kits of phentolamine, an alpha-adrenergic antagonist intended for monotherapy or used as a combination therapy in the treatment of patients having presbyopia, mydriasis, and/or other ocular complications. The phentolamine, instilled topically, preferably is supplied as aqueous ophthalmic preparation.	Presbyopia, mydriasis, and other ocular problem	[123]
US7795203B2	Method for topical treatment of eye disease, composition, and device for said treatment (2010)	The invention pertaining to aqueous ophthalmic preparation comprised of a *N*-acetylcarnosine derivative or a pharmacologically acceptable salt of *N*-acetylcarnosine with cellulose imparting intraocular absorption of the said compound. The patent further disclosed a hydrogel contact lens, an ocular insert made of polymer for topical instillation containing *N*-acetylcarnosine and *N*-acetylcarnosine derivatives.	Eye diseases (corneal problems, glaucoma, presbyopia, and blurred vision)	[124]
KR20200000395A	Eye composition containing cyclosporine and a method of preparing the same (2020)	The current invention disclosed a composition of an ophthalmic nanoemulsion that contained cyclosporine, in a highly solubilized state as an active ingredient, and their stability improved by incorporating drug in a nonaqueous vehicle, an emulsifier, and an aqueous vehicle. The nanoemulsion established desired particle size of 100 nm and narrow particle distribution.	Dry eye syndrome, Conjunctivitis, and tingling sensation in the eye	[125]
KR101008189B1	Ophthalmic nanoemulsion composition containing cyclosporin for the treatment of dryeyesyndrome (2011)	An ophthalmic composition having cyclosporine (0.01–0.1%), propylene glycol monocarprylate (0.1–2.5%). poloxamer (0.5–15%) glycerine, (0.1–10%), chitosan or carbomer (0.1–5%) and purified water (67.4–98.5%). The method for preparing the composition Follows dissolving the drug in a propylene glycol monocarprylate gradeI solution, thereafter dissolving poloxamer, glycerin, and chitosan or carbomer in purified water to obtain a grade II solution, and finally mixing the I and II step solution while adjusting pH by 7.2–7.6.	Dryeyesyndrome	[126]
KR20200053205A	A surfactant-free type ophthalmic nanoemulsion composition and the manufacturing method thereof	This invention provides a composition of eye drop nanoemulsion and their manufacturing aspects thereof. The composition hascyclosporine, a solubilizing agent, a stabilizer, and a solvent. The solubilizing agent established a nanoemulsion with a droplet size of 20 nm or less. The invention of nanoemulsion eye drop improves stability and patient compliance.	Dry eye syndrome	[127]
US6956057B2	EP4 agonists as agents for lowering intraocular pressure (2005)	The invention relates to an active compound, EP4 agonist meant for the treatment of ocular hypertension or glaucoma. The efficacy of the invention was proved by administering in animal models possessing ocular hypertension or glaucoma.	Ocular hypertension or glaucoma	[128]
US8980839B2	Topical aqueous nanomicellar, ophthalmic solutions and uses thereof (2015)	The invention disclosed herein a method and composition of formulations for topical instillation. The composition of the formulation may include a polyoxyl lipid (fatty acid) and/or a polyalkoxylated alcohol and nanomicelles. In a further embodiment, methods herein describe the treatment procedures for preventing ocular diseases.	Ocular diseases	[129]
US5945121A	Liposome eye drops (1999)	The present invention of liposomal eye drops having taurine, glucose, and inorganic salts, with adjusted pH ranging 5.5–8.0. The osmotic pressure of formulation is maintained in between 250 and 450 mOsm. Further, the taurine and inorganic salts are entrapped in lipid vesicles viz., the liposomes.	Mitigation of dry eye problem	[130]
WO2017152129A2	Treatment of glaucoma and/or retinal diseases (2017)	The invention provided herein is an ophthalmic formulationand methods of its use. The embodiments of the formulations may include a polyoxyl lipid or fatty acid, and/or a polyalkoxylated alcohol in nanomicelles. It also includes the method of treating or preventing ocular diseases or conditions.	Glaucoma and retinal diseases	[131]
WO2018095429A1	Use of gold cluster or gold-cluster-containing substance in preparation of drugs for preventing and/or treating glaucoma (2018)	The disclosed invention relates to the application of gold-cluster-bearing substances in the drug composition for the treatment and mitigation of glaucoma.	Glaucoma	[132]
CN110664757A	Nanocrystalline eye drop, preparation method, and application thereof (2020)	The invention provides preparation of nanocrystalline eye drop and their application. The composition of eye drop is single soluble (chitosan or hyaluronic acid) and double (poloxamer and tween) soluble macromolecule, and a drug targeting on endothelial growth factor receptor or a platelet growth factor receptor. The interaction among double and single soluble macromolecule encloses the target drug as nanocrystal and preserves the nanocrystal stability. The medicament enabled the penetration of various barriers in the eye and entered the vitreous humor by permeation or passive targeting via intercellular space/pinocytosis and could achieve effective drug concentration in the cul-de-sac cavity.	Congenital macular degeneration	[133]
US9295693B2	Bifunctional copolymer use for ophthalmic and other topical and local applications (2016)	This invention studied a water-soluble graft copolymer poly(L-Lysine)-graft-poly(ethylene glycol) with good surface featuresin terms of biological surfaces. The composition was further sterically stabilized with PEG coatings and made compatible with biological surfaces. The composition is useful in dry eye syndrome and contact lens intolerance therapy.	Dry eye syndrome	[134]
US20200237925A1	Polypeptide eye absorption enhancer and use thereof	This pharmaceutical preparation relates to synthetic cell penetration, i.e., peptides. The peptide has the potential ability to permeate the ocular tissues, with no ocular toxicity reported. The peptides actas absorption enhancers and are delivered through a noninvasive route for intraocular delivery and thus improve ocular drug bioavailability. The invention is a promised alternative to intraocular injection and patient compliance for the treatment of intraocular diseases.	Intraocular and fundus diseases.	[135]
CN109906075A	Liposome corticosteroid for the locally injecting in inflammation lesion or region (2019)	Thisinvention relates to corticosteroid liposome for local use and inflammatory region on ocular tissues. The composition of liposomes having uncharged lipid or Pegylated liposome for the treatment of inflammation lesion with the one-time applicationin two weeks.	Inflammatory lesion in eye	[136]
US6995186B2	Olopatadine formulations for topical administration	Topical formulations of olopatadine for the treatment of allergic or inflammatory disorders of the eye and nose are disclosed. The aqueous formulations contain approximately 0.17–0.62% (*w*/*v*) of olopatadine and an amount of polyvinylpyrrolidone or polystyrene sulfonic acid sufficient to enhance the physical stability of the formulations.	Inflammatory eye disorder	[137]

## Data Availability

Not applicable.

## References

[1] Gholizadeh S., Wang Z., Chen X., Dana R., Annabi N. (2021). Advanced nanodelivery platforms for topical ophthalmic drug delivery. Drug Discov. Today.

[2] Nayak K., Misra M. (2020). Triamcinolone Acetonide-Loaded PEGylated Microemulsion for the Posterior Segment of Eye. ACS Omega.

[3] Bachu R.D., Chowdhury P., Al-Saedi Z.H.F., Karla P.K., Boddu S.H.S. (2018). Ocular Drug Delivery Barriers—Role of Nanocarriers in the Treatment of Anterior Segment Ocular Diseases. Pharmaceutics.

[4] Almeida H., Amaral M.H., Lobao P., Lobo J.M.S. (2014). In situ gelling systems: A strategy to improve the bioavailability of ophthalmic pharmaceutical formulations. Drug Discov. Today.

[5] Gorantla S., Rapalli V.K., Waghule T., Singh P.P., Dubey S.K., Saha R.N., Singhvi G. (2020). Nanocarriers for ocular drug delivery: Current status and translational opportunity. RSC Adv..

[6] Huang D., Chen Y.S., Rupenthal I.D. (2018). Overcoming ocular drug delivery barriers through the use of physical forces. Adv. Drug Deliv. Rev..

[7] Achouri D., Alhanout K., Piccerelle P., Andrieu V. (2012). Recent advances in ocular drug delivery. Drug Dev. Ind. Pharm..

[8] Wu Y., Liu Y., Li X., Kebebe D., Zhang B., Ren J., Lu J., Li J., Du S., Liu Z. (2019). Research progress of in-situ gelling ophthalmic drug delivery system. Asian J. Pharm. Sci..

[9] Joseph R.R., Venkatraman S.S. (2017). Drug delivery to the eye: What benefits do nanocarriers offer?. Nanomedicine.

[10] Weng Y., Liu J., Jin S., Guo W., Liang X., Hu Z. (2016). Nanotechnology-based strategies for treatment of ocular disease. Acta Pharm. Sin. B.

[11] Gaudana R., Ananthula H.K., Parenky A., Mitra A.K. (2010). Ocular Drug Delivery. AAPS J..

[12] Subrizi A., del Amo E.M., Korzhikov-Vlakh V., Tennikova T., Ruponen M., Urtti A. (2019). Design principles of ocular drug delivery systems: Importance of drug payload, release rate, and material properties. Drug Discov. Today.

[13] Nayak K., Choudhari M.V., Bagul S., Chavan T.A., Misra M., Chappel E. (2020). Ocular drug delivery systems. Developments in Biomedical Engineering and Bioelectronics, Drug Delivery Devices and Therapeutic Systems.

[14] Kang-Mieler J.J., Rudeen K.M., Liu W., Mieler W.F. (2020). Advances in ocular drug delivery systems. Eye.

[15] Thornit D.N., Vinten C.M., Sander B., Lund-Andersen H., La Cour M. (2010). Blood–Retinal Barrier Glycerol Permeability in Diabetic Macular Edema and Healthy Eyes: Estimations from Macular Volume Changes after Peroral Glycerol. Investig. Opthalmology Vis. Sci..

[16] Tavakoli S., Peynshaert K., Lajunen T., Devoldere J., del Amo E.M., Ruponen M., De Smedt S.C., Remaut K., Urtti A. (2020). Ocular barriers to retinal delivery of intravitreal liposomes: Impact of vitreoretinal interface. J. Control. Release.

[17] Adrianto M.F., Annuryanti F., Wilson C.G., Sheshala R., Thakur R.R.S. (2021). In vitro dissolution testing models of ocular implants for posterior segment drug delivery. Drug Deliv. Transl. Res..

[18] Chen P., Chen H., Zang X., Chen M., Jiang H., Han S., Wu X. (2013). Expression of Efflux Transporters in Human Ocular Tissues. Drug Metab. Dispos..

[19] Zhang T., Xiang C.D., Gale D., Carreiro S., Wu E.Y., Zhang E.Y. (2008). Drug Transporter and Cytochrome P450 mRNA Expression in Human Ocular Barriers: Implications for Ocular Drug Disposition. Drug Metab. Dispos..

[20] Vaka S.R.K., Sammeta S.M., Day L., Murthy S.N. (2008). Transcorneal Iontophoresis for Delivery of Ciprofloxacin Hydrochloride. Curr. Eye Res..

[21] Moiseev R.V., Morrison P.W.J., Steele F., Khutoryanskiy V.V. (2019). Penetration Enhancers in Ocular Drug Delivery. Pharmaceutics.

[22] Opitz D.L., Harthan J.S. (2012). Review of Azithromycin Ophthalmic 1% Solution (AzaSite®) for the Treatment of Ocular Infections. Ophthalmol. Eye Dis..

[23] Tajika T., Isowaki A., Sakaki H. (2011). Ocular Distribution of Difluprednate Ophthalmic Emulsion 0.05% in Rabbits. J. Ocul. Pharmacol. Ther..

[24] Liu Y., Lin X., Tang X. (2009). Lipid emulsions as a potential delivery system for ocular use of azithromycin. Drug Dev. Ind. Pharm..

[25] Shen J., Gan L., Zhu C., Zhang X., Dong Y., Jiang M., Zhu J., Gan Y. (2011). Novel NSAIDs ophthalmic formulation: Flurbiprofen axetil emulsion with low irritancy and improved anti-inflammation effect. Int. J. Pharm..

[26] Agrahari V., Mandal A., Agrahari V., Trinh H.M., Joseph M., Ray A., Hadji H., Mitra R., Pal D., Mitra A.K. (2016). A comprehensive insight on ocular pharmacokinetics. Drug Deliv. Transl. Res..

[27] Yellepeddi V., Palakurthi S. (2016). Recent Advances in Topical Ocular Drug Delivery. J. Ocul. Pharmacol. Ther..

[28] Mazet R., Yaméogo J.B.G., Wouessidjewe D., Choisnard L., Gèze A. (2020). Recent Advances in the Design of Topical Ophthalmic Delivery Systems in the Treatment of Ocular Surface Inflammation and Their Biopharmaceutical Evaluation. Pharmaceutics.

[29] Scoper S.V., Kabat A.G., Owen G.R., Stroman D.W., Kabra B.P., Faulkner R., Kulshreshtha A.K., Rusk C., Bell B., Jamison T. (2008). Ocular distribution, bactericidal activity and settling characteristics of TobraDex ST ophthalmic suspension compared with TobraDex ophthalmic suspension. Adv. Ther..

[30] Baranowski P., Karolewicz B., Gajda M., Pluta J. (2014). Ophthalmic Drug Dosage Forms: Characterisation and Research Methods. Sci. World J..

[31] Patel A., Cholkar K., Agrahari V., Mitra A.K. (2013). Ocular drug delivery systems: An overview. World J. Pharmacol..

[32] Benson H.A., Grice J.E., Mohammed Y., Namjoshi S., Roberts M.S. (2019). Topical and Transdermal Drug Delivery: From Simple Potions to Smart Technologies. Curr. Drug Deliv..

[33] Civiale C., Licciardi M., Cavallaro G., Giammona G., Mazzone M.G. (2009). Polyhydroxyethyl aspartamide-based micelles for ocular drug delivery. Int. J. Pharm..

[34] Musumeci T., Bucolo C., Carbone C., Pignatello R., Drago F., Puglisi G. (2013). Polymeric nanoparticles augment the ocular hypotensive effect of melatonin in rabbits. Int. J. Pharm..

[35] Zhang L., Li Y., Zhang C., Wang Y., Song C. (2009). Pharmacokinetics and tolerance study of intravitreal injection of dexame-thasone-loaded nanoparticles in rabbits. Int. J. Nanomed..

[36] Chi H., Gu Y., Xu T., Cao F. (2017). Multifunctional organic–inorganic hybrid nanoparticles and nanosheets based on chitosan derivative and layered double hydroxide: Cellular uptake mechanism and application for topical ocular drug delivery. Int. J. Nanomed..

[37] Kassem M., Rahman A.A., Ghorab M.M., Ahmed M., Khalil R.M. (2007). Nanosuspension as an ophthalmic delivery system for certain glucocorticoid drugs. Int. J. Pharm..

[38] Boddeda B., Boddu P., Avasarala H., Jayanti V. (2015). Design and Ocular Tolerance of Flurbiprofen Loaded Nanosuspension. Pharm. Nanotechnol..

[39] Santos G.A.D., Ferreira-Nunes R., Dalmolin L.F., Ré A.C.D.S., Anjos J.L.V., Mendanha S.A., Aires C.P., Lopez R.F.V., Cunha-Filho M., Gelfuso G.M. (2020). Besifloxacin liposomes with positively charged additives for an improved topical ocular delivery. Sci. Rep..

[40] Natarajan J.V., Chattopadhyay S., Ang M., Darwitan A., Foo S., Zhen M., Koo M., Wong T.T., Venkatraman S.S. (2011). Sustained release of an anti-glaucoma drug: Demonstration of efficacy of a liposomal formulation in the rabbit eye. PLoS ONE.

[41] Rajala A., Wang Y., Zhu Y., Ranjo-Bishop M., Ma J.X., Mao C., Rajala R.V. (2014). Nanoparticle-assisted targeted delivery of eye-specific genes to eyes significantly improves the vision of blind mice in vivo. Nano Lett..

[42] Law S.L., Huang K.J., Chiang C.H. (2000). Acyclovir-containing liposomes for potential ocular delivery. Corneal penetration and ab-sorption. J. Cont. Rel..

[43] Vandamme T.F., Brobeck L. (2005). Poly (amidoamine) dendrimers as ophthalmic vehicles for ocular delivery of pilocarpine nitrate and tropicamide. J. Control. Release.

[44] Lancina M.G., Singh S., Kompella U.B., Husain S., Yang H. (2017). Fast Dissolving Dendrimer Nanofiber Mats as Alternative to Eye Drops for More Efficient Antiglaucoma Drug Delivery. ACS Biomater. Sci. Eng..

[45] Phua J.L., Hou A., Lui Y.S., Bose T., Chandy G.K., Tong L., Venkatraman S., Huang Y. (2018). Topical Delivery of Senicapoc Nanoliposomal Formulation for Ocular Surface Treatments. Int. J. Mol. Sci..

[46] Sun K., Hu K. (2021). Preparation and Characterization of Tacrolimus-Loaded SLNs in situ Gel for Ocular Drug Delivery for the Treatment of Immune Conjunctivitis. Drug Des. Dev. Ther..

[47] Noriaki N., Misa M., Saori D., Hiroko O., Hiroshi S., Naoki Y. (2020). An in situ Gelling System Based on Methylcellulose and Tranilast Solid Nanoparticles Enhances Ocular Residence Time and Drug Absorption into the Cornea and Conjunctiva. Front Bioeng and Biotech..

[48] Rebibo L., Tam C., Sun Y., Shoshani E., Badihi A., Nassar T., Benita S. (2021). Topical tacrolimus nanocapsules eye drops for therapeutic effect enhancement in both anterior and posterior ocular inflammation models. J. Cont. Rel..

[49] Robinson R., Viviano S.R., Criscione J.M., Williams C.A., Jun L., Tsai J.C., Lavik E.B. (2011). Nanospheres delivering the EGFR TKI AG1478 promote optic nerve regeneration: The role of size for intraocular drug delivery. ACS Nano.

[50] Giannavola C., Bucolo C., Maltese A., Paolino D., Vandelli M.A., Puglisi G., Lee V.H.L., Fresta M. (2003). Influence of Preparation Conditions on Acyclovir-Loaded Poly-d,l-Lactic Acid Nanospheres and Effect of PEG Coating on Ocular Drug Bioavailability. Pharm. Res..

[51] Khames A., Khaleel M., El-Badawy M., El-Nezhawy A. (2019). Natamycin solid lipid nanoparticles—sustained ocular delivery system of higher corneal penetration against deep fungal keratitis: Preparation and optimization. Int. J. Nanomed..

[52] Yadav M., Schiavone N., Guzman-Aranguez A., Giansanti F., Papucci L., Perez De Lara M.J., Singh M., Kaur I.P. (2020). Atorvastatin-loaded solid lipid nanoparticles as eye drops: Proposed treatment option for age-related macular degeneration (AMD). Drug Deliv. Transl. Res..

[53] Paiva M.R.B., Andrade G.F., Dourado L.F.N., Castro B.F.M., Fialho S.L., Sousa E.M.B., Silva-Cunha A. (2020). Surface functionalized mesoporous silica nanoparticles for intravitreal application of tacrolimus. J. Biomater. Appl..

[54] Kim J.H., Kim J.H., Kim K.W., Kim M.H., Yu Y.S. (2009). Intravenously administered gold nanoparticles pass through the blood–retinal barrier depending on the particle size, and induce no retinal toxicity. Nanotechnology.

[55] Mitra M., Kandalam M., Rangasamy J., Shankar B., Maheswari U.K., Swaminathan S., Krishnakumar S. (2013). Novel epithelial cell adhesion mol-ecule antibody conjugated polyethyleneimine-capped gold nanoparticles for enhanced and targeted small interfering RNA delivery to retinoblastoma cells. Mol. Vis..

[56] Gade S.K., Nirmal J., Garg P., Venuganti V.V.K. (2020). Corneal delivery of moxifloxacin and dexamethasone combination using drug-eluting mucoadhesive contact lens to treat ocular infections. Int. J. Pharm..

[57] Kim J., Chauhan A. (2008). Dexamethasone transport and ocular delivery from poly (hydroxyethyl methacrylate) gels. Int. J. Pharm..

[58] Gulsen D., Li C.C., Chauhan A. (2005). Dispersion of DMPC liposomes in contact lenses for ophthalmic drug delivery. Curr. Eye Res..

[59] Lee S.S., Hughes P., Ross A.D., Robinson M.R. (2010). Biodegradable implants for sustained drug release in the eye. Pharm. Res..

[60] Choonara Y.E., Pillay V., Danckwerts M.P., Carmichael T.R., du Toit L.C. (2010). A review of implantable intravitreal drug delivery technologies for the treatment of posterior segment eye diseases. J. Pharm. Sci..

[61] Sheshala R., Hong G.C., Yee W.P., Meka V.S., Thakur R.R.S. (2018). In situ forming phase-inversion implants for sustained ocular delivery of triamcinolone acetonide. Drug Deliv. Transl. Res..

[62] Mirzaeei S., Taghe S., Asare-Addo K., Nokhodchi A. (2021). Polyvinyl Alcohol/Chitosan Single-Layered and Polyvinyl Alcohol/Chitosan/Eudragit RL100 Multi-layered Electrospun Nanofibers as an Ocular Matrix for the Controlled Release of Ofloxacin: An In Vitro and In Vivo Evaluation. AAPS PharmSciTech.

[63] Xue J., Xie J., Liu W., Xia Y. (2017). Electrospun Nanofibers: New Concepts, Materials, and Applications. Accounts Chem. Res..

[64] Khalil I., Ali I.H., El-Sherbiny I.M. (2019). Noninvasive biodegradable nanoparticles-in-nanofibers single-dose ocular insert: In vitro, ex vivo and in vivo evaluation. Nanomedicine.

[65] Taghe S., Mirzaeei S., Alany R., Nokhodchi A. (2020). Polymeric Inserts Containing Eudragit^®^ L100 Nanoparticle for Improved Ocular Delivery of Azithromycin. Biomedicines.

[66] Singh R.R.T., Tekko I., McAvoy K., McMillan H., Jones D., Donnelly R. (2016). Minimally invasive microneedles for ocular drug delivery. Expert Opin. Drug Deliv..

[67] Jiang J., Gill H.S., Ghate D., McCarey B.E., Patel S.R., Edelhauser H.F., Prausnitz M.R. (2007). Coated microneedles for drug delivery to the eye. Investig. Ophthalmol. Vis. Sci..

[68] Jiang J., Moore J.S., Edelhauser H.F., Prausnitz M.R. (2009). Intra-scleral drug delivery to the eye using hollow microneedles. Pharm. Res..

[69] Than A., Liu C., Chang H., Duong P.K., Cheung C.M.G., Xu C., Wang X., Chen P. (2018). Self-implantable double-layered micro-drug-reservoirs for efficient and controlled ocular drug delivery. Nat. Commun..

[70] Akhter M.H., Ahmad A., Ali J., Mohan G. (2014). Formulation and Development of CoQ10-Loaded s-SNEDDS for Enhancement of Oral Bioavailability. J. Pharm. Innov..

[71] Kalam M.A., Alshamsan A., Aljuffali I.A., Mishra A.K., Sultana Y. (2016). Delivery of gatifloxacin using microemulsion as vehicle: Formulation, evaluation, transcorneal permeation and aqueous humor drug determination. Drug Deliv..

[72] Perminaite K., Marksa M., Ivanauskas L., Ramanauskiene K. (2021). Preparation of Ophthalmic Microemulsions Containing Lithuanian Royal Jelly and Their Biopharmaceutical Evaluation. Processes.

[73] Junnuthula V., Boroujeni A.S., Cao S., Tavakoli S., Ridolfo R., Toropainen E., Ruponen M., van Hest J., Urtti A. (2021). Intravitreal Polymeric Nanocarriers with Long Ocular Retention and Targeted Delivery to the Retina and Optic Nerve Head Region. Pharmaceutics.

[74] Alami-Milani M., Zakeri-Milani P., Valizadeh H., Salehi R., Jelvehgari M. (2017). Preparation and evaluation of PCL-PEG-PCL micelles as potential nanocarriers for ocular delivery of dexamethasone. Iran. J. Basic Med. Sci..

[75] Vaishya R.D., Gokulgandhi M., Patel S., Minocha M., Mitra A.K. (2014). Novel Dexamethasone-Loaded Nanomicelles for the Intermediate and Posterior Segment Uveitis. AAPS PharmSciTech.

[76] Mehra N., Aqil M., Sultana Y. (2021). A grafted copolymer-based nanomicelles for topical ocular delivery of everolimus: Formulation, characterization, ex-vivo permeation, in-vitro ocular toxicity, and stability study. Eur. J. Pharm. Sci..

[77] Patel S., Garapati C., Chowdhury P., Gupta H., Nesamony J., Nauli S., Boddu S.H. (2015). Development and Evaluation of Dexamethasone Nanomicelles with Potential for Treating Posterior Uveitis After Topical Application. J. Ocul. Pharmacol. Ther..

[78] Xu X., Sun L., Zhou L., Cheng Y., Cao F. (2019). Functional chitosan oligosaccharide nanomicelles for topical ocular drug delivery of dexamethasone. Carbohydr. Polym..

[79] Diebold Y., Calonge M. (2010). Applications of nanoparticles in ophthalmology. Prog. Retin. Eye Res..

[80] Kesavan K., Balasubramaniam J., Kant S., Singh P.N., Pandit J.K. (2011). Newer approaches for optimal bioavailability of ocularly de-livered drugs: Review. Curr. Drug Deliv..

[81] Ahmad M.Z., Rizwanullah, Ahmad J., Alasmary M.Y., Akhter H., Abdel-Wahab B.A., Warsi M.H., Haque A. (2021). Progress in nanomedicine-based drug delivery in designing of chitosan nanoparticles for cancer therapy. Int. J. Polym. Mater..

[82] Bhatta R.S., Chandasana H., Chhonker Y.S., Rathi C., Kumar D., Mitra K., Shukla P.K. (2012). Mucoadhesive nanoparticles for prolonged ocular delivery of natamycin: In vitro and pharmacokinetics studies. Int. J. Pharm..

[83] Yu A., Shi H., Liu H., Bao Z., Dai M., Lin D., Lin D., Xu X., Li X., Wang Y. (2019). Mucoadhesive dexamethasone-glycol chitosan nanoparticles for ophthalmic drug delivery. Int. J. Pharm..

[84] Correa Z.M., Berry J.L. (2016). Retinoblastoma. American Academy of Ophthalmology. https://www.aao.org/pediatric-center-detail/retinoblastoma-2016.

[85] Bhavsar D., Subramanian K., Sethuraman S., Krishnan U.M. (2016). Management of retinoblastoma: Opportunities and challenges. Drug Deliv..

[86] Akhter M.H., Beg S., Tarique M., Malik A., Afaq S., Choudhry H., Hosawi S. (2021). Receptor-based targeting of engineered nanocarrier against solid tumors: Recent progress and challenges ahead. Biochim. Biophys. Acta BBA-Gen. Subj..

[87] Ahmad J., Ahmad M.Z., Akhter M.H. (2020). Surface-Engineered Cancer Nanomedicine: Rational Design and Recent Progress. Curr. Pharm. Des..

[88] Akhter M.H., Ahsan M.J., Rahman M., Anwar S., Rizwanullah M. (2020). Advancement in Nanotheranostics for Effective Skin Cancer Therapy: State of the Art. Curr. Nanomed..

[89] Akhter M.H., Amin S. (2017). An Investigative Approach to Treatment Modalities for Squamous Cell Carcinoma of Skin. Curr. Drug Deliv..

[90] Akhter M.H., Rizwanullah M., Ahmad J., Amin S., Ahmad M.Z., Minhaj A., Mujtaba A., Ali J. (2021). Molecular Targets and Nanopar-ticulate Systems Designed for the Improved Therapeutic Intervention in Glioblastoma Multiforme. Drug Res..

[91] Akhter M.H., Khalilullah H., Gupta M., Alfaleh M.A., Alhakamy N.A., Riadi Y., Shadab M. (2021). Impact of Protein Corona on the Bi-ological Identity of Nanomedicine: Understanding the Fate of Nanomaterials in the Biological Milieu. Biomedicines.

[92] Qu W., Meng B., Yu Y., Wang S. (2018). Folic acid-conjugated mesoporous silica nanoparticles for enhanced therapeutic efficacy of topotecan in retina cancers. Int. J. Nanomed..

[93] Yan R., Xu L., Wang Q., Wu Z., Zhang H., Gan L. (2021). Cyclosporine A Nanosuspensions for Ophthalmic Delivery: A Comparative Study between Cationic Nanoparticles and Drug-Core Mucus Penetrating Nanoparticles. Mol. Pharm..

[94] Kausar H., Mujeeb M., Ahad A., Moolakkadath T., Aqil M., Ahmad A., Akhter H. (2019). Optimization of ethosomes for topical thymoquinone delivery for the treatment of skin acne. J. Drug Deliv. Sci. Technol..

[95] Lai S., Wei Y., Wu Q., Zhou K., Liu T., Zhang Y., Jiang N., Xiao W., Chen J., Liu Q. (2019). Liposomes for effective drug delivery to the ocular posterior chamber. J. Nanobiotechnol..

[96] Shen H.-H., Chan E.C., Lee J.H., Bee Y.-S., Lin T.-W., Dusting G.J., Liu G.S. (2015). Nanocarriers for treatment of ocular neovascularization in the back of the eye: New vehicles for ophthalmic drug delivery. Nanomedicine.

[97] Gupta S.K., Velpandian T., Dhingra N., Jaiswal J. (2000). Intravitreal pharmacokinetics of plain and liposome-entrapped fluconazole in rabbit eyes. J. Ocul. Pharmacol. Ther..

[98] Zhang R., He R., Qian J., Guo J., Xue K., Yuan Y.F. (2010). Treatment of experimental autoimmune uveoretinitis with intravitreal in-jection of tacrolimus (FK506) encapsulated in liposomes. Investig. Ophthalmol. Vis. Sci..

[99] Keam S.J., Scott L.J., Curran M.P. (2003). Verteporfin: A review of its use in the management of subfoveal choroidal neovascularisation. Drugs.

[100] Soiberman U., Kambhampati S.P., Wu T., Mishra M.K., Oh Y., Sharma R., Wang J., Al Towerki A.E., Yiu S., Stark W.J. (2017). Subconjunctival injectable dendrimer-dexamethasone gel for the treatment of corneal inflammation. Biomaterials.

[101] Torres-Luna C., Fan X., Domszy R., Hu N., Wang N.S., Yang A. (2020). Hydrogel-based ocular drug delivery systems for hydrophobic drugs. Eur. J. Pharm. Sci..

[102] Wang P., Chu W., Zhuo X., Zhang Y., Gou J., Ren T., He H., Yin T., Tang X. (2017). Modified PLGA–PEG–PLGA thermosensitive hydrogels with suitable thermosensitivity and properties for use in a drug delivery system. J. Mater. Chem. B.

[103] Chen Y., Li Y., Shen W., Li K., Yu L., Chen Q., Ding J. (2016). Controlled release of liraglutide using thermogelling polymers in treatment of diabetes. Sci. Rep..

[104] Akhter M.H., Rizwanullah, Ahmad J., Ahsan M.J., Mujtaba A., Amin S. (2017). Nanocarriers in advanced drug targeting: Setting novel paradigm in cancer therapeutics. Artif. Cells Nanomed. Biotechnol..

[105] Katzer T., Chaves P., Bernardi A., Pohlmann A., Guterres S., Beck R. (2014). Prednisolone-loaded nanocapsules as ocular drug delivery system: Development, in vitrodrug release and eye toxicity. J. Microencapsul..

[106] Li F., Hurley BLiu Y., Leonard B., Griffith M. (2012). Controlled Release of Bevacizumab through Nanospheres for Extended Treatment of Age-Related Macular Degeneration. Open Ophthalmol. J..

[107] Akhter M.H., Madhav N.S., Ahmad J. (2018). Epidermal growth factor receptor based active targeting: A paradigm shift towards advance tumor therapy. Artif. Cells Nanomed. Biotechnol..

[108] Müller R.H., Lucks J.S. (1996). Arzneistoffträger Aus Festen Lipidteilchen-Feste Lipid Nanosphären (SLN). European Patent.

[109] Badilli U., Gumustas M., Uslu B., Ozkan S.A., Grumezescu A.M. (2018). Lipid-based nanoparticles for dermal drug delivery. Organic Materials as Smart Nanocarriers for Drug Delivery.

[110] Ali H., Singh S.K. (2016). Biological voyage of solid lipid nanoparticles: A proficient carrier in nanomedicine. Ther. Deliv..

[111] Nair A., Shah J., Al-Dhubiab B., Jacob S., Patel S., Venugopala K., Morsy M., Gupta S., Attimarad M., Sreeharsha N. (2021). Clarithromycin Solid Lipid Nanoparticles for Topical Ocular Therapy: Optimization, Evaluation and In Vivo Studies. Pharmaceutics.

[112] Sun Y., Huffman K., Freeman W.R., Sailor M.J., Cheng L. (2020). Intravitreal safety profiles of sol-gel mesoporous silica microparticles and the degradation product (Si(OH)_4_). Drug Deliv..

[113] Masse F., Ouellette M., Lamoureux G., Boisselier E. (2018). Gold nanoparticles in ophthalmology. Med. Res. Rev..

[114] Li Q., Ma C., Ma Y., Ma Y., Mao Y., Meng Z. (2021). Sustained bimatoprost release using gold nanoparticles laden contact lenses. J. Biomater. Sci. Polym. Ed..

[115] Gupta H., Aqil M. (2012). Contact lenses in ocular therapeutics. Drug Discov. Today.

[116] Lp J. (2016). A Summary of Recent Advances in Ocular Inserts and Implants. J. Bioequiv. Bioavailab..

[117] Sun Y.J., Lin C.-H., Wu M.-R., Lee S.H., Yang J., Kunchur C.R., Mujica E.M., Chiang B., Jung Y.S., Wang S. (2021). An intravitreal implant injection method for sustained drug delivery into mouse eyes. Cell Rep. Methods.

[118] Xin S., Zeng Z., Zhou X., Luo W., Shi X., Wang Q., Deng H., Du Y. (2017). Recyclable Saccharomyces cerevisiae loaded nanofibrous mats with sandwich structure constructing via bio-electrospraying for heavy metal removal. J. Hazard. Mater..

[119] Grimaudo M.A., Concheiro A., Alvarez-Lorenzo C. (2020). Crosslinked Hyaluronan Electrospun Nanofibers for Ferulic Acid Ocular Delivery. Pharmaceutics.

[120] Donnelly R.F., Raj Singh T.R., Woolfson A.D. (2010). Microneedle-based drug delivery systems: Microfabrication, drug delivery, and safety. Drug Deliv..

[121] Thakur R.R.S., Tekko I., Al-Shammari F., Ali A.A., McCarthy H., Donnelly R. (2016). Rapidly dissolving polymeric microneedles for minimally invasive intraocular drug delivery. Drug Deliv. Transl. Res..

[122] Meyer A. (2021). Methods and Compositions for Daily Ophthalmic Administration of Phentolamine to Improve Visual Performance. U.S. Patent.

[123] Pitlick W.H., Meyer A.R., Sooch M., Charizanis K., Hoffmann B. (2021). Methods and Compositions for Treatment of Presbyopia, My-Driasis, and Other Ocular Disorders. U.S. Patent.

[124] Babizhayev M.A. (2004). Method for Topical Treatment of Eye Disease and Composition and Device for Said Treatment. U.S. Patent.

[125] Junyeop L., Jae S.Y., Sang-rok R. (2020). Eye Composition Containing a Cyclosporine and a Method of Preparing the Same.

[126] Seon-kyung, Gwang-ho C., Jin-woo N. (2011). OphthalmicNano-Emulsion Composition Containing Cyclosporin for the Treatment of Dry-Eye-Syndrome.

[127] Chul-hwan K., Hyun-seop N., Hye-min K., Da-hye S. (2020). A Surfactant-Free Type Ophthalmic Nano-Emulsion Composition, and the Manufacturing Method Thereof.

[128] Woodward D.F., Krauss A.H., Burk R.M., Holoboski M., Posner M.F. (2005). EP4 agonists as agents for lowering intraocular pressure. U.S. Patent.

[129] Mitra A.K., Weiss S.L. (2015). Topical Aqueous Nanomicellar, Ophthalmic Solutions and Uses Thereof. U.S. Patent.

[130] Kato M., Ohtsuki T., Egami F., Tsunoda K. (1999). Liposome Eye Drops. U.S. Patent.

[131] Weiss S.L. (2017). Treatment of Glaucoma and/or Retinal Diseases.

[132] (2018). Use of Gold Cluster or Gold Cluster-Containing Substance in Preparation of Drug for Preventing and/or Treating Glaucoma.

[133] Qing D. (2020). Nanocrystalline eye drop, preparation method and application thereof.

[134] Cooper E.R., Kleinman D.M., Loxley A., Mitchnick M. (2016). Bi-Functional Co-Polymer Use for Ophthalmic and Other Topical and Local Applications. U.S. Patent.

[135] Wei G., Jiang K., Lu W., Liu C., Tai L., Gao X. (2020). Polypeptide Eye Absorption Enhancer and Use Thereof. U.S. Patent.

[136] (2019). Liposome Corticosteroid for the Locally Injecting in Inflammation Lesion or Region. https://patents.google.com/patent/CN109906075A/en.

[137] Castillo E.J., Han W.W., Zhang H., Bhagat H.G., Singh O.N., Bullock J.P., Dixit S.C. (2006). Olopatadine formulations for topical admin-istration. U.S. Patent.

[138] Meyer A. (2021). Aqueous Ophthalmic Solutions of Phentolamine and Medical Uses Thereof. U.S. Patent.

